# How reliance on Spanish-language social media predicts beliefs in false political narratives amongst Latinos

**DOI:** 10.1093/pnasnexus/pgae442

**Published:** 2024-11-19

**Authors:** Marisa Abrajano, Marianna Garcia, Aaron Pope, Robert Vidigal, Joshua A Tucker, Jonathan Nagler

**Affiliations:** Department of Political Science, UC San Diego, La Jolla, CA 92093, USA; Department of Political Science, UC San Diego, La Jolla, CA 92093, USA; Center for Social Media and Politics, New York University, New York, NY 10012, USA; Center for Social Media and Politics, New York University, New York, NY 10012, USA; Center for Social Media and Politics, New York University, New York, NY 10012, USA; Department of Politics, New York University, New York, NY 10012, USA

## Abstract

False political narratives are nearly inescapable on social media in the United States. They are a particularly acute problem for Latinos, and especially for those who rely on Spanish-language social media for news and information. Studies have shown that Latinos are vulnerable to misinformation because they rely more heavily on social media and messaging platforms than non-Hispanic whites. Moreover, fact-checking algorithms are not as robust in Spanish as they are in English, and social media platforms put far more effort into combating misinformation on English-language media than Spanish-language media, which compounds the likelihood of being exposed to misinformation. As a result, we expect that Latinos who use Spanish-language social media to be more likely to believe in false political narratives when compared with Latinos who primarily rely on English-language social media for news. To test this expectation, we fielded the largest online survey to date of social media usage and belief in political misinformation of Latinos. Our study, fielded in the months leading up to and following the 2022 midterm elections, examines a variety of false political narratives that were circulating in both Spanish and English on social media. We find that social media reliance for news predicts one’s belief in false political stories, and that Latinos who use Spanish-language social media have a higher probability of believing in false political narratives, compared with Latinos using English-language social media.

**Significance Statement**

Despite existing research identifying an abundance of misinformation on Spanish-language social media, we are unaware of any systematic research examining its consequences on Latinos’ political attitudes in the United States. We address this research gap by testing empirically if self-reported exposure to Spanish-language social media predicts whether Latinos find misinformation circulating on social media to be credible. We test this claim by fielding an original online panel survey that, to the best of our knowledge, is the largest survey conducted of US-based Latinos examining the relationship between social media usage and their respective political beliefs. We find that Latinos who use Spanish-language social media for news are more likely to believe these false narratives relative to Latinos who use English-language social media, even when controlling for primary language spoken at home.

## Introduction

Concerns over misinformation are a pressing issue in politics, particularly as it relates to the types of political information and news to which individuals are exposed (1–8). A common way that misinformation enters our everyday lives is via popular social media platforms, such as Facebook, YouTube, and Twitter/X. Media reports reveal that the amount of political misinformation is far greater on Spanish-language vs. English-language social media platforms (9–11); a 2022 Axios headline referred to it as “The Spanish-language misinformation crisis (12).” Moreover, a study by Nielsen reveals that Latinos are more vulnerable to misinformation because they rely more heavily on social media and messaging platforms than do non-Hispanic whites (13). The study also suggests that “Latinos are avid users of these apps because of the trust and intimacy they offer and their unique role within the community to connect people to family and friends both in the United States and abroad” ((13), p. 11). Another source of misinformation stems from the fact that traditional fact-checking algorithms face difficulties monitoring content in Spanish, given that the content can be a combination of formal Spanish, colloquial Spanish, and Spanglish^a^ (10, 13). Finally, foreign sources have also contributed to the misinformation circulating on Spanish-language social media, especially as it relates to COVID-19 (14). This misinformation could have significant effects on US Latinos: a recent study by Velez et al. (15) found that Latinos—and especially those who are Spanish-speakers—were susceptible to changing their beliefs when exposed to misinformation in an experimental context.

Such concerns are even more pressing considering that the 2024 US elections are quickly approaching, and there is no doubt that social media platforms will be replete with mis- and disinformation targeting Latinos, especially Spanish speakers. A study by Equis Research reported that during the 2020 US elections, YouTube videos targeting Latinos contained numerous false statements in hopes of convincing them to support Trump (16). Despite reports of an abundance of misinformation on Spanish-language social media, we are not aware of any systematic research examining its consequences on Latinos’ political attitudes and beliefs. We fill this gap in the literature by testing empirically the following hypothesis:

Latinos who use Spanish-language social media will be more likely than those using English-language social media to find false stories to be credible.

We test this claim by fielding an original online panel survey that, to the best of our knowledge, is the largest study conducted of Latinos examining the relationship between social media usage and beliefs. Understanding how Latinos’ information sources affect their awareness of current events is crucial, given that Latinos are about 25% of the US population, and nearly 40% of Latinos are foreign-born, which means that many are still learning about the ins and outs of American politics (17–19). Latinos also continue to exhibit politically heterogeneous preferences and are “up for grabs” in many key swing states (17, 20, 21); as such, it is crucial for them to have access to information environments that allow them to make informed decisions.

## Results

We asked respondents whether they considered seven political narratives to either be true or false (see the Materials and methods section for specific question wordings). Overall, we find empirical support for our hypothesis. Across the seven false narratives, the pooled first difference estimate for the effect of relying on Spanish-language social media for news rather than English-language social media for news on believing in a false narrative to be 11 percentage points.^b^ This indicates that Latinos who use Spanish-language social media for news are 11 percentage points more likely to believe in false political narratives than those who use English-language social media for news. This effect size suggests social media (and the language of that social media in particular) exerts a substantively meaningful effect on a Latino’s belief in political misinformation.

**Fig. 1.**
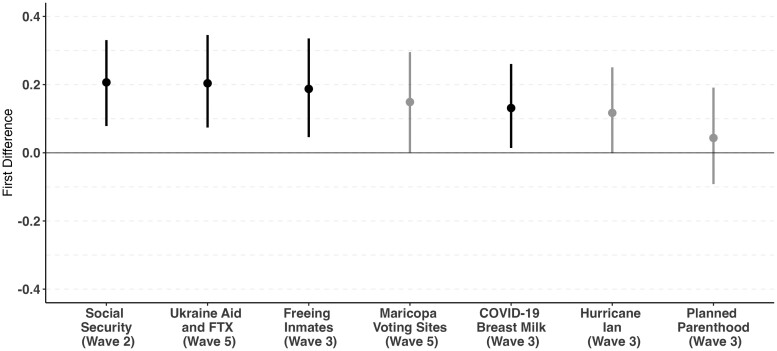
First difference estimates for false political narratives. Estimates represent the average first difference in predicted probabilities between Latino respondents who click on links to read political news stories on Spanish-language social media in comparison to those who do so on English-language social media. Error bars represent 95% CIs.

Figure 1 presents the first difference estimates showing the average difference in predicted probabilities of believing each of our seven false political narratives for a Latino individual who accesses news on Spanish-language social media (3 or more days per week) compared with a Latino individual who accesses English-language social media for news. The change in probability for 6 of the 7 questions shows that Latinos who access Spanish-language social media for news were between 11 and 20 percentage points more likely to believe each of these false narratives than Latinos who use English-language social media. Notably, this result holds even when controlling for the primary language spoken at home.

The questions that we asked were wide-ranging in scope and subject matter, yet the estimates are generally consistent in showing that Latinos accessing news through Spanish-language social media have a higher probability in believing these political events to have occurred, relative to Latinos using English-language social media for news. And six of the seven individual estimates are significant at (P-value<0.10), with four significant at traditional levels (P-value<0.05).^c^ We also note that the effect sizes are relatively consistent across these six false narratives. While Cortina and Rottinghaus (20) find that partisanship—namely identifying as a Republican and also being a Trump supporter—is an important predictor of believing in conspiracy theories among Latinos, partisanship emerges as a statistically significant predictor for only two of the false political narratives: (i) Venezuela purposely freeing inmates to send to the US Border and (ii) Planned Parenthood closing clinics across the country. The magnitude of the partisanship effect is also smaller for usage of social media to access news.^d^

### Robustness check

Thus far, our results lend support to our hypothesis linking Latinos’ Spanish-language social media usage with increased probability of believing in false political narratives. However, past research has raised concerns over acquiescence bias (22, 23), the tendency to agree with survey questions, particularly on studies focusing on conspiracy theories or misconceptions (24, 25). To test for the possibility of acquiescence bias, we included multiple survey items asking respondents about true political narratives related to similar topics. As with the false political narrative questions, respondents were given the option to state whether each of the true political narratives below were either “true” or “false” alongside belief certainty. We designed these survey items based on current events that occurred during the time period prior to and following the 2022 midterm elections as well as those stories that are relevant to the Latino population. In total, we asked about five true narratives:

1. The US government will provide $2 Million in aid to Cuba to support recovery efforts following Hurricane Ian. [Foreign Aid and Cuba]
2. Some studies published in peer-review journals have observed a rare correlation between the COVID-19 vaccine and myocarditis, an inflammation of the heart muscle that can cause chest pain and shortness of breath, especially in young men. [COVID-19 and Myocarditis]
3. President Biden has promised that if the Democrats retain control of the House and Senate, the first bill he will send to Congress next year will federally protect abortion rights. [Abortion Rights]
4. The Department of Homeland Security will expand Title 42 expulsion in order to turn away Venezuelans that present at the United States–Mexico Border seeking asylum. [Venezuelans Asylum Ban]
5. Recently elected New York Representative George Santos made numerous dubious and false claims about his biography, work history, and financial status while running for office. [George Santos Claims]

**Fig. 2.**
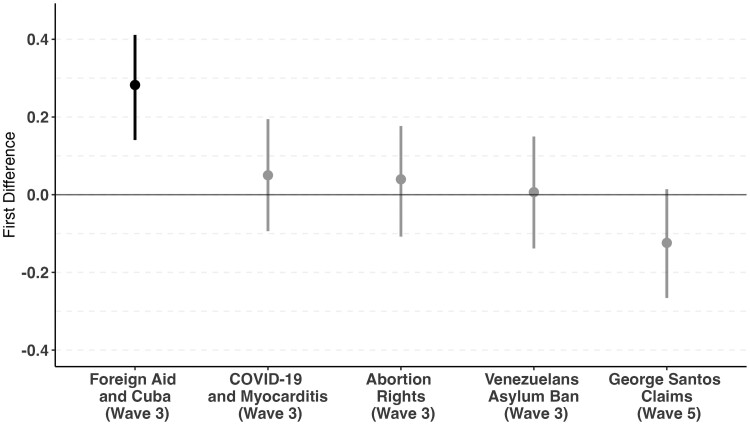
First difference estimates for true political narratives. Estimates represent the first difference in predicted probabilities between Latino respondents who click on links to read political news stories on Spanish-language social media vs. those who do so on English-language social media. Error bars represent 95% CIs.

We use the same model specification as with the false political narratives, and once again we calculate first difference estimates to determine the average difference in predicted probabilities of Latinos who use Spanish-language social media for news regularly compared with Latinos who regularly use English-language social media for news. The results are presented in Fig. 2; only one of the five estimates is statistically significant. Moreover, the pooled mean of the five estimates only slightly differs from zero. As such, these findings should largely alleviate concerns that acquiescence bias could be driving our previous results. Relative to Latinos who use English-language social media, those relying on Spanish-language social media for news are more likely to believe that foreign aid to Cuba is true. Given that we do not find that social media usage consistently increases the likelihood of believing in true stories, we are confident that our survey respondents are actually making the effort to evaluate each of these political narratives.

## Discussion

Our study offers the first evidence that the widespread misinformation inundating millions of Latinos on social media leads to factually inaccurate political beliefs, which could have important downstream consequences for democracy in America. We are able to link the widespread belief that misinformation is a problem on Spanish-language media to the actual *outcome* of interest: increased belief in misinformation. By fielding what we believe to be the largest original online panel survey of Latinos to understand their social media usage and political beliefs, we confirm our hypothesis that Latinos who turn to Spanish-language social media for news are more likely to believe in false political narratives than Latinos who use English-language social media for news. Across an array of false narratives pertaining to COVID-19, natural disaster relief, immigration policy, electoral fraud, and foreign aid, the relationship between social media news source and beliefs in political falsehoods persists.

Altogether, our research offers evidence of the deleterious effects of false political information on Latinos’ knowledge of important news and current events. Given that many Latinos who rely on Spanish-language information sources are recent entrants into the US political process (17, 18), deciphering whether their information sources are providing accurate information, in addition to the overall effort required to engage in politics, is no small feat.^e^

These findings raise several concerns regarding whether Spanish-speaking Latinos can be manipulated and deceived in US politics to a greater extent than groups of voters who rely on more reliable information sources. Political elites, interest groups, and other stakeholders, especially those competing in the upcoming 2024 Presidential election, may therefore target Spanish-speaking Latinos with specific false narratives in order to gain their support. Such was the case in the 2020 Presidential election (10), and there is little doubt that they will do so again.

While social media companies could make efforts to be more rigorous in their fact-checking efforts, the proliferation of misinformation on an array of Spanish-language social media platforms may make such efforts a challenge. That being the case, studying how Latinos’ information sources affect their awareness of current events and policy is crucial, given their growing prominence in the population nationwide (Latinos are projected to be 25% of the US population by 2050) as well as their sizable presence in key swing states such as Florida and Arizona (17, 20, 27). More research on ways to combat these false narratives amongst the Latino electorate is therefore needed.

## Materials and methods

We test our hypothesis, which predicts that Latinos who rely on Spanish-language social media for news will exhibit a higher likelihood of believing in false political narratives when compared with Latinos using English-language sources, by fielding what we believe to be the largest original online panel survey of Latino social media usage and their political beliefs. Given our expectations of differential exposure to political misinformation by language, we recruited a large sample of Latinos consisting of approximately equal numbers of English-dominant, bilingual, and Spanish-dominant respondents, as well as a large sample of non-Hispanic whites.^f^

These respondents were recruited by collecting a sample of US Facebook and Instagram users through paid advertisements via an online ads campaign; we note that this approach is consistent past the research studying social media and politics (28–30). The ads, which were offered in English and Spanish, promoted a survey about “news consumption” and offered a monetary incentive for participating in the survey. The campaign was conducted using Meta Ads that were optimized using Meta’s Pixel, a piece of code that tracks landing page views to optimize ad delivery to the target population and maximize landing page views.^g^

Our research study protocol was approved by New York University’s Institutional Review Board (reference number: IRB-FY2020-4,463). Informed consent was obtained from all participants. We conducted a total of five survey waves, with the first campaign fielded from 2022 March 24 to July 25 and the last wave fielded from 2023 February 8 to 27. Wave 2 was in the field from 2022 September 22 to October 23; wave 3 was in the field from 2022 October 8 to November 8, and wave 5 was in the field from 2023 February 8 to 28.^h^ Altogether, the sample of Latinos for this analysis is *N* = 1,116.^i^ The data are weighted through a post-stratification raking algorithm to fall within the margin of error of the adult population in the 2019 Census American Community Survey (ACS) 5-year data file for age, gender, education, and US region.^j^

The primary dependent variables of interest are seven survey items that were based on false political narratives circulating during the 2022 election cycle. These survey items were fielded in wave 2, wave 3, and wave 5 surveys. We selected these political narratives as they were fact-checked by one of three sources: AP News, Univision, or Factchequeado.^k^ The responses were coded as belief or nonbelief in the false political narratives.^l^ The responses to these questions also included a nonopinion response (“unsure”), given the possibility for acquiescence bias (24, 25). We treated “unsure” responses as a lack of belief in false information.^m^ The seven false political narratives that we asked about were:

1. Some people are claiming that US border patrol agents have been giving out social security numbers to immigrants who cross into the United States at the southern border without authorization. [Social Security]
2. US aid to Ukraine was laundered back to the Democratic Party through the failed cryptocurrency exchange firm FTX. [Ukraine Aid and FTX]
3. The Department of Homeland Security has confirmed that Venezuela is purposely freeing inmates and sending them to the United States–Mexico Border. [Freeing Inmates]
4. The only voting sites in Arizona that experienced issues with tabulating ballots on election day during the 2022 midterm elections were conservative areas in Arizona’s Maricopa County. [Maricopa Voting Sites]
5. A new study shows that getting the COVID-19 vaccine can make breast milk dangerous to infants. [COVID-19 Breast Milk]
6. Vice President Kamala Harris said that Hurricane Ian relief will be distributed based on race, with communities of color receiving aid first. [Hurricane Ian]
7. After the *Dobbs vs. Jackson Women’s Health* decision that overturned the right to have an abortion, the majority of Planned Parenthood clinics have closed down across the country. [Planned Parenthood]

The empirical strategy we use to test our hypothesis is to estimate probit models on belief in each of the seven narratives as the outcome variable to determine whether, all else equal, Latinos who rely on Spanish-language social media exhibit a higher likelihood of believing in false political narratives when compared with Latinos using English social media sources. The primary explanatory variable of interest is an interaction term that captures whether one uses Spanish-language social media as a source of news and does so for 3 days or more on a weekly basis. We estimate a separate model for each of the seven narratives to allow for effects of consuming Spanish language media to vary across the different false narratives.

To illustrate the substantive effects of these estimated probit coefficients, we calculated first differences (31) in predicted probabilities between Latinos who use Spanish-language social media for news vs. Latinos who use English-language social media for news. A battery of control variables pertaining to demographics (e.g. gender, education, income, birthplace) and partisanship asked in the first survey wave were also included in the probit model.^n^ Note that we are also conditioning on Latinos’ reported home language-use in these models.

## Supplementary Material

pgae442_Supplementary_Data

## Data Availability

The underlying survey data, code, and all Supplementary material will be made available on the Harvard Dataverse repository.

## Notes

a A mix of English and Spanish: https://www.merriam-webster.com/dictionary/Spanglish.

b

P-value<0.05 , CI [0.01, 0.20]. Table D13 in Appendix D presents the probit estimates for a pooled model with individual level fixed-effects.

c Both the Hurricane Ian and the Maricopa estimate just miss significance at (P-value<0.05) but are significant at (P-value<0.10).

d See Appendix D, Figs. D1 and D2, for the first difference estimates of partisanship of each false political narratives among Latinos.

e Recent research on immigrant socialization also indicates the importance of social relationships in this process (26).

f We classified respondents as Latino if: (i) they self-identified as Hispanic or Latino/a/x when asked what racial or ethnic group best described them or (ii) they chose to take the survey in Spanish. While the choice to take a survey in Spanish does not suggest that someone either identifies as Hispanic or is of Hispanic/Latino origin, follow-up questions with respondents suggested that the overwhelming majority of persons taking the survey in Spanish were of Hispanic origin.

g Refer to Appendix A for a detailed description of panelist recruitment, participation/response rates, and data cleaning.

h We exclude wave 4 from this analysis since no misinformation questions were asked in this wave.

i The sample size for each wave (wave 1, wave 2, wave 3, and wave 5) varies as it is contingent on the dependent variable being analyzed. See Table A2 in Appendix A for wave-to-wave participation rates for racial and language groups.

j Refer to Tables B1–B4 in Appendix B, which compare demographics of our online sample to the ACS.

k See Appendix E for exact question wording, the source of each question, and more details on the methodology for selecting false narratives.

l Respondents indicated on a 5-point scale, ranging from 1 indicating “very sure is true” to a 5 indicating “very sure is false”; we also included an“unsure” option, which was the midpoint of the scale. In only wave 2, we employed a 7-point scale, ranging from “definitely true” to “definitely false.”

m In Appendix D, we perform a robustness check and find that omitting respondents who said unsure left the conclusions unchanged.

n Refer to Appendix C on the coding of variables used in the probit analyses, and for details on the calculation of first differences. Control variables and social media language were asked in wave 1; social media use was asked in wave 2.
