# Supplementary material for: How reliance on Spanish-language social media predicts beliefs in false political narratives amongst Latinos
Source: PNAS Nexus. 2024 Nov 19;3(11):pgae442. doi: 10.1093/pnasnexus/pgae442 (PMC11561907; doi:10.1093/pnasnexus/pgae442)
Supplement: pgae442_Supplementary_Data [file pgae442_supplementary_data.pdf]

APPENDIX A: Survey Recruitment and Data Cleaning

Recruitment: Strategy, Campaign, and Data Quality

Our goal was to recruit a sample of 4,000 respondents (with a large oversampling of Latinos) to take multiple surveys prior to and immediately following the 2022 Midterm Elections. Within our Latino sample, we aimed for a mix of respondents that were English-dominant, Spanish-dominant, and bilingual in their language preferences.

Recruitment (Wave 1) took place from March 24, 2022, through July 25, 2022. Our sample was predominantly recruited using advertisements on Facebook and Instagram through Meta Business Ads platform. Our ads, which ran in Spanish and English, highlighted the compensation for taking the survey and noted that participation would: "Help Improve Online Information Integrity and Political Knowledge in the United States." Survey compensation was $5 from March 24, 2022, through May 11, 2022, and was increased to $10 from May 12, 2022 to July, 25, 2022 to incentivize participation. Compensation was sent via Tango card, a website that allows for the purchase of a variety of gift cards. Payments were made no later than three weeks after the completion of each survey.

Consistent with past research, we encountered difficulties in recruiting Latino respondents, especially those who reported being Spanish-dominant (Brown, 2015; Sha et al., 2017). This could not be remedied directly using Meta Ads, because as of January 19, 2022, Meta removed the ability to target ads based on racial or Ethnic groups (Meta, 2022). To overcome this issue, we experimented with several proxies for targeting potential Latino respondents with our ads, including targeting users with Latino interests,[1] users in zip codes with large Latino populations (zip codes that are greater than 70% Latino), and users that are interested in Latino countries and their culture.[2] However, the most effective targeting strategy we used is called a "Look-a-Like" audience, a feature in Meta's ad platform that allowed us to target individuals similar to our previous Latino respondents (balanced on gender).

We also attempted to recruit respondents via text message. On May 15, 2022, we contracted

[1]Latin American music, Bachata (music), Copa Libertadores, Univision Communications, Banda (music), Cumbia, CONCACAF, Telenovela, Reggaeton, Medalla Light, Merengue (dance) or Liga MX.

[2]Mexico, Cuba, Argentina, Bolivia, Brazil, Chile, Columbia, Costa Rica, Dominican Republic, Ecuador, El Salvador, Guatemala, Honduras, Nicaragua, Panama, Paraguay, Peru, Puerto Rico, Uruguay, and Venezuela.

the firm Stones Phones to send text messages with survey links to a random sample 50,000 potential Latino respondents stratified on gender[3] (60% Male and 40% female) that were purchased through the data firm L2. In total there were only 48 valid responses, so these reflect a very small portion of our total respondents (0.14%).

In order to oversample Latinos respondents, we implemented screening based on demographic characteristics. From March 24, 2022 through April 10 2022, we recruited subjects from all demographic groups. From April 11, 2022 through May 3, 2022, we only accepted responses from individuals that self-identified as Republicans, Independents, or Hispanic/Latinx in the English survey or those that responded to a Spanish ad. From May 4, 2022, through July 25, 2022, we only accepted responses from individuals that self-identified as Hispanic/Latinx in the English survey or responded to a Spanish ad. We also had two pauses to recruitment. We temporarily paused English language ads from April 2, 2022 through April 11, 2022, to analyze our data quality and implement screener questions; and from May 30, 2022 to June 15, 2022, we paused all ads in preparation for out attempt to recruit via text message using Stones Phones. On July 25, 2022, we completed our recruitment.

Table A1: Survey Participation & Response Rates

**Meta Ads Recruitment**		**Text Message Recruitment**	
Reach (Estimated by Meta)	2,134,990	Contact List	50,000
Link Clicks	74,730	Individuals Successfully Contacted	49,393
Survey Starts	19,280	Survey Starts	187
Passed Demographics Screener	10,823	Passed Demographics Screener	69
Survey Completions	9,691	Survey Completions	48
Valid Survey Completions	3,330	Valid Survey Completions	48
Survey Start Rate	0.21%	Survey Start Rate	0.14%
Participation Rate	0.16%	Response Rate	0.10%

Note: The difference between Survey Completions and Valid Survey completions, is whether a response passed all data quality cleaning steps (please contact authors for more details on the data cleaning process.)

In Table A1, we provide a summary of our recruitment, as well as the response rate for the text message recruitment and the participation rate for the Meta Ads recruitment [4]. As our text message recruitment was done using a random sample, we calculate response rate using the American Association for Public Opinion Research's (AAPOR) guidance for SMS or text message surveys (AAPOR,

[3]This was done in an attempt to help address gender imbalance in the sample.

[4]American Association for Public Opinion Research advises the use of the term participation rate (rather than response rate) for non-probability samples.

2023). We provide the calculation for "Response Rate 1" (also known as minimum response rate) detailed in the AAPOR's Standard Definitions (2023). AAPOR does not offer guidance on calculating participation rate for online non-probability samples, and cautions against calculating them (2023). To calculate our Meta Ads response rates we have adapted the calculation i AAPOR's guidance for probability-based online surveys; we use the following formula:

$$\text{Participation Rate} = \frac{\text{Valid Survey Completions}}{\text{Reach} - (\text{Screened for Demographics} + \text{Responses Cleaned for Data Quality})}$$

AAPOR does not offer guidance on calculating survey start rates; we have calculated ours using the following formula:

$$\text{Survey Start Rate} = \frac{\text{Survey Starts} - (\text{Screened for Demographics} + \text{Cleaned for Data Quality})}{\text{Reach or Contact List} - (\text{Screened for Demographics} + \text{Cleaned for Data Quality})}$$

The participation rate for our Meta Ads Campaign was approximately 0.16%. While this is very low compared to probability samples, we follow AAPOR's guidance on cautioning against making comparisons across recruitment methods (AAPOR, 2023). One complicating factor for comparisons of other recruitment methods is that the denominator in our calculation is: the "reach" of the ads minus individuals known to be ineligible. The reach of the ads is estimated by Meta, and it is unclear how many people actually processed the ads while scrolling on social media, as it is a less attention-grabbing invitation than a letter, phone call or text message. Additionally, it is unclear how many people who saw our ads were eligible to participate, as we were recruiting a large oversample of Latinos. The variability of targeting is also born-out in the fact that approximately 44% of those that started our survey were screened by our demographic criteria. While there has been a significant increase in the number of studies relying on respondents recruited on Facebook, very few disclose the reach of the ads. Even articles aimed at helping researchers understand and improve recruitment online often do not disclose the reach of the ads (Antoun et al., 2016; Boas et al., 2020; Zhang et al., 2020).

Studies that did disclose the reach of their ads found lower or comparable participation rates to our study (Bunge et al., 2017; Neundorf and Öztürk, 2023; Schneider and Harknett, 2022). Bunge et al. (2017) used Facebook Ads to recruit a sample of 540 U.S.-based individuals for an online

study for Spanish- and English-speaking smokers. The total reach of their ads was 1,034,038, which makes their participation rate approx 0.05%. Schneider and Hacket (2022) used Facebook to survey employees of a number of large U.S. retails chains (Gap, Old Navy, Target, etc.). Their recruitment indicated that their ads reached 3,270,228 potential participants for 17,828 survey completions (participation rate = 0.55%). While neither are perfect comparisons, they do offer valuable context, and show that our participation rate is comparable or higher than other studies that disclosed their ad's reach. We present the reach of our ads and other descriptive statistics in the interest of transparency.

In Table A2, we provide the participation rates for each survey we offered beyond the first wave. We note that these are actually the retention rates for each wave. The overall retention rate for each wave is approximately 60%. The wave-to-wave participation rate is lower for Latino respondents than it is for "white/other race" groups. We also consistently observe the greatest participation rates from English-language respondents, followed by bilingual and then our Spanish-language respondents. However, across all groups, the wave-to-wave participation rate is relatively consistent across surveys.

We report the demographic distributions of our respondents in Tables B1 thru B4, and compare them to the United States voting age population as reported by the Census Bureau. We note that for gender, education, and age - we achieve reasonable coverage with the exception of low-education individuals: only 4% of our Latino respondents have less than a high-school education. Thus while inferences drawn based on education would be suspect (we are having to heavily up-weight a small sample), we are confident that we have recruited an otherwise demographically representative sample. We of course can *not* be sure that our respondents are representative on all other characteristics. But as our goal here is to compare the relationship between an observed behavior (consumption of Spanish language media online) and belief in a series of misinformation narratives – we believe our findings safely generalize to a large set of the Latino population.

Ensuring Survey Data Quality

Across survey waves we took both *ex ante* and *ex post* precautions to ensure data integrity. Our *ex ante* procedures included a preliminary VPN and IP address screen that would not allow anyone

Table A2: Survey Participation in Each Wave by Racial and Language Groups

	**Wave 1**	**Wave 2**	**Wave 3**	**Wave 4**	**Wave 5**
White (Survey Completions)	882	652	663	669	675
White Participation Rate	.	73.92%	75.17%	75.85%	76.53%
Latinos (Survey Completions)	2152	1178	1097	1117	1181
Latino Participation Rate	.	54.74%	50.98%	51.91%	54.88%
EL Social Media (Survey Completions)	803	506	475	479	509
EL Social Media Participation Rate	.	63.01%	59.15%	59.65%	63.39%
SL Social Media (Survey Completions)	360	168	156	168	186
SL Social Media Participation Rate	.	46.67%	43.33%	46.67%	51.67%
BI Social Media (Survey Completions)	981	501	462	464	482
BI Social Media Participation Rate	.	51.07%	47.09%	47.30%	49.13%
Other Race (Survey Completions)	344	258	258	261	274
Other Race Participation Rate	.	75.00%	75.00%	75.87%	79.65%
Total Completions	3378	2088	2018	2047	2130
Total Participation Rate	.	61.81%	59.74%	60.60%	63.06%

Note: "EL Social Media" means English-language social media user. "SL Social Media" means Spanish-language social media user. "BI Social Media" means bilingual social media user. Participation rate is calculated by dividing the number of survey completions by the number of survey invitations sent. The number of survey invitations sent is equal to the number of completions in Wave 1. There were 8 Latino respondents who indicated that when they read news on social media they read in "Neither" English or Spanish, so they are excluded in any social media language group comparisons.

to complete the survey if it was detected they were using a VPN or taking the survey from outside the United States. Ahler et al. (2019) found that crowd sourced respondents tended to circumvent location requirements, use multiple devices from the same IP address, and blacklisted IP addresses.

Our *ex post* procedures integrated the processes outlined by past research (Waggoner et al., 2019; Griffin et al., 2022; Trejo et al., 2022). In Table A3 we detail how many respondents were cleaned from each racial and language group during our*ex post* data cleaning procedures. These include removing individuals that met any of the following criteria in the recruitment survey:

1. Had a Google reCaptcha score lower than 0.5 or provided an answer to a 'honey pot' question[5](Griffin et al., 2022)
2. Provided multiple responses from the same email address[6] (Griffin et al., 2022)
3. Provided multiple responses from the same IP addresses[7] (Griffin et al., 2022)
4. Completed the survey in less than 5 minutes (Griffin et al., 2022)
5. Completed the survey in more than 60 minutes (Griffin et al., 2022)
6. Responded to a question about zip code with an answer that did not correspond to their self-reported state (Griffin et al., 2022)
7. Responded to a question about state with an answer that did not corresponded to survey meta data (Waggoner et al., 2019)
8. VPN or proxy server identified in *ex post* check, using an additional two services (Waggoner et al., 2019)

Following the guidance of Griffin et al. (2022) and Trejo et al. (2022), we took the additional step of having three coders independently review open-ended responses to flag responses that appear suspicious. In contrast to closed-ended questions, open-ended questions are more sensitive for detecting fraudulent responses. For example, indicators of low response quality are copying and

[5]'Honey pot' questions are hidden using JavaScript so they invisible to from human respondents, but visible to bots. Therefore, any response to such questions indicates the respondent is a bot.

[6]For respondents with multiple submissions using the same email address, we kept their first submission if they answered the following demographic questions the same in subsequent surveys: age, state of residence, gender, race, party ID, voter status.

[7]Unlike duplicate emails, all submissions by duplicate IP addresses were dropped.

pasting text from the question stem; random typing; repeating the same exact text across open-ended probes; or non sequitur answers that had nothing to do with the topic of the question (Griffin et al., 2022; Trejo et al., 2022). Any respondent who was flagged by two out of the three coders was removed. This procedure was also helpful for identifying respondents that were ineligible, such as non-Spanish-speakers posing as Spanish-speakers (Trejo et al., 2022).

Finally, we repeated our *ex ante* screen for IP Address outside the U.S/VPNs, and our *ex post* checks for bots, VPNs, and Proxy Servers in Waves 2-5, and retroactively removed any respondents who failed any data quality check in any survey.

Table A3: Data Cleaning Across Racial and Language Groups

	Whites	Latinos	SL Social Media	EL Social Media	BI Social Media	Other Race	Total
Total Survey Completions	2311	5970	1055	2353	2529	1458	9739
Respondent Reported Being < 18 (after screener)	3	3	0	3	0	1	7
Google RCaptcha Score <.5 or answered 'honey pot' question	103	367	58	135	172	119	589
Duplicate Emails	87	360	42	142	173	83	530
Duplicate IP Address	334	1089	172	460	445	261	1684
Completed Survey in < 5 Min	5	7	4	1	2	113	125
Completed Survey in > 60 Min	9	94	33	15	46	9	112
Self-Report Zip Code and State Don't Match	127	178	49	71	58	174	479
Self-Reported State and Geolocation Metadata Don't Match	502	950	169	386	390	218	1670
Proxy Server Identified in Post Hoc Check	156	117	22	54	39	72	345
Qualitative Answer Review	16	169	56	71	42	7	192
Failed Any Quality Checks in Surveys 2 – 5	87	484	90	212	181	57	628
Total Clean Sample	882	2152	360	803	981	344	3378
Total Cleaned	1429	3818	695	1550	1548	1114	6361
% Cleaned from Sample*	61.83	63.95	65.88	65.87	61.21	76.41	65.31

Note: "EL Social Media" means English-language social media user. "SL Social Media" means Spanish-language social media user. "BI Social Media" means bilingual social media user.The percentage cleaned is calculated by dividing the total number of cleaned responses (Total Cleaned) by the total number of surveys completed (Total Survey Completions).

APPENDIX B: Survey Data Weights and Marginals

Survey Data Weighting

We construct our population target weights for our survey weights based on the American Commmunity Survey (ACS). The ACS collects survey data continuously, nearly every day of the year and then aggregates the results over the specific time period. We use the ACS 5-year estimate due to its' higher precision on population targets and we compute individual and aggregated weights for the following variables: *Gender; Age; Highly Educated (6 categories); Region.* Tables B1, B2, B3, and B4 below presents the ACS population targets (ACS columns) and our sample estimates (CSIP columns) before weighting. We use the R package *svydesign* to create the weights using the raking method.[1]

Table B1: Gender - ACS Targets and CSIP Unweighted Frequencies

	All Respondents		Latinos	
Gender	ACS	CSIP	ACS	CSIP
Men	49%	44%	49%	44%
Women	51%	56%	51%	56%

Note: ACS values are population targets from the Census Bureau's American Community Survey, CSIP values are from the survey conducted by the authors.

Table B2: Highly Educated (6 categories) - ACS Targets and CSIP Unweighted Frequencies

	All Respondents		Latinos	
Highly Educated (6 categories)	ACS	CSIP	ACS	CSIP
No High School	10%	3%	25%	4%
High School	28%	16%	32%	16%
Some College	17%	19%	17%	19%
Associate's Degree	10%	12%	8%	14%
College Degree	22%	32%	13%	32%
Postgraduate	13%	18%	5%	15%

Note: ACS values are population targets from the Census Bureau's American Community Survey, CSIP values are from the survey conducted by the authors.

[1] See https://www.pewresearch.org/methods/2018/01/26/for-weighting-online-opt-in-samples-what-matters-most/ for a discussion of weighting of opt-in samples.

Table B3: Age - ACS Targets and CSIP Unweighted Frequencies

	All Respondents		Latinos	
Age	ACS	CSIP	ACS	CSIP
18-24	10%	17%	17%	17%
25-34	15%	32%	23%	37%
35-44	17%	22%	21%	23%
45-54	23%	12%	17%	13%
55-64	22%	8%	12%	7%
65-99	13%	6%	11%	3%

Note: ACS values are population targets from the Census Bureau's American Community Survey, CSIP values are from the survey conducted by the authors.

Table B4: Region - ACS targets and CSIP estimates

	All Respondents		Latinos	
Region	ACS	CSIP	ACS	CSIP
Northeast	17%	25%	18%	24%
Midwest	21%	12%	14%	9%
West	38%	25%	39%	27%
South	24%	38%	29%	40%

Note: ACS values are population targets from the Census Bureau's American Community Survey, CSIP values are from the survey conducted by the authors.

APPENDIX C: Multivariable Model Parameter Estimates

Coding of Variables Used in Probit Analyses

Female: Coded as "1" if respondent selects female as their gender, "0" otherwise.

Evangelical: Coded as "1" if respondent selects evangelical as their religious affiliation, "0" otherwise.

Democrat: Coded as "1" if respondent selects Democrat as their partisan affiliation, "0" otherwise.

Republican: Coded as "1" if respondent selects Republican as their partisan affiliation, "0" otherwise.

Highly Educated: Categorical variable that ranges from "1" less than HS degree to "6" Postgraduate degree.

Age 18-34: Coded as "1" if respondent is between the ages of 18-34, "0" otherwise.

Age 35-44: Coded as "1" if respondent is between the ages of 35-44, "0" otherwise.

Age 45-64: Coded as "1" if respondent is between the ages of 45-64, "0" otherwise.

Income (greater than $60k): Coded as "1" if respondent is greater than $60,000, "0" otherwise.

Born in the U.S.: Coded as "1" if respondent reports being born in the U.S., "0" otherwise.

Cuban: Coded as "1" if respondent reports being of Cuban origin, "0" otherwise.

Mexican: Coded as "1" if respondent reports being of Mexican origin, "0" otherwise.

Puerto Rican: Coded as "1" if respondent reports being of Puerto Rican origin, "0" otherwise.

Social Media Accounts: Continuous variable ranging from 1-14; it captures the number of social media sites that a respondent reports using.

Social Media News Language (Bilingual): Coded as "1" if respondent reports getting their news from social media in both English and Spanish, "0" otherwise.

Social Media News Language (Spanish): Coded as "1" if respondent reports getting their news from social media in Spanish, "0" otherwise.

Bilingual Home: Coded as "1" if respondent reports speaking both Spanish and English equally at home, "0" otherwise.

Spanish Home: Coded as "1" if respondent reports speaking only Spanish/more Spanish than English at home, "0" otherwise.

3 days or more Social Media Use: Categorical variable that ranges from "1" for respondents who report never clicking on links to read political news stories on social media to "6" for respondents who do so on an everyday basis.

Fox News Viewer: Coded as "1" if respondent reports following Fox News as a source for political news and information, "0" otherwise.

Social Media News Language (Bilingual) * Social Media Use (3 days or more): Coded as "1" if respondent is bilingual and reports clicking on links to read political news stories on social media at least 3 days a week or more, "0" otherwise.

Social Media News Language (Spanish) * Social Media Use (3 days or more): Coded as "1" if respondent is Spanish-dominant and reports clicking on links to read political news stories on social media at least 3 days a week or more, "0" otherwise.

First differences calculation

Evaluating one's theoretical expectations regarding the effect of changes in the independent variable(s) of interest on the dependent variable is the primary goal of observational studies. We calculate predicted probabilities for each of two values of Social Media News Language (English and Spanish) for all respondents, while holding all other variables constant. First differences reported are the values of the difference between those estimated probabilities, averaged over all respondents (average effect approach). We use the DAMisc package in R, which uses a parametric bootstrap to generate simulated confidence bounds for predicted probabilities and their differences. The confidence intervals produced are at the 95% level.

Tables C1 to C7 present the coefficients from the probit models from which we calculate the first differences presented in Figure 1 in the main text (false stories). Tables C8 to C12 present the coefficients from the probit models from which we calculate the first differences presented in Figure 2 in the main text (true stories).

Table C1: Probit Estimates for Wave 2 False Story on U.S. border patrol agents have been giving out social security numbers to immigrants (Latinos)

Wave 2	Social Security
(Intercept)	−1.12*** (0.30)
Gender (female)	0.02 (0.09)
Evangelical	0.21 (0.14)
Democrat	−0.11 (0.11)
Republican	0.01 (0.13)
Highly Educated (6 categories)	−0.09* (0.04)
Age 18-34	0.18 (0.22)
Age 35-44	0.51* (0.22)
Age 45-64	0.45 (0.24)
Income (greater than $60k)	0.12 (0.10)
Born in the U.S.	0.80*** (0.12)
Cuban	−0.33 (0.20)
Mexican	−0.62*** (0.11)
Puerto Rican	−0.47** (0.17)
Social Media Accounts	−0.03 (0.02)
Social Media News Language (Bilingual)	0.01 (0.15)
Social Media News Language (Spanish)	0.06 (0.19)
Bilingual home	0.07 (0.12)
Spanish home	0.18 (0.13)
Social Media Use (3 days or more)	−0.14 (0.14)
Fox News Viewer	0.32** (0.10)
Social Media News Language (Bilingual) * Social Media Use (3 days or more)	0.24 (0.20)
Social Media News Language (Spanish) * Social Media Use (3 days or more)	0.64* (0.26)
McFadden R^2	0.13
Percent Correctly Predicted	78.11%
AIC	1100.45
BIC	1215.54
Log Likelihood	−527.23
Deviance	1054.45
Number of Observations	1101

Note: the numbers between parenthesis represent standard errors. $^{***}p < 0.001$; $^{**}p < 0.01$; $^{*}p < 0.05$

Table C2: Probit Estimates for Wave 3 False Story on Vice President Kamala Harris said that Hurricane Ian relief will be distributed based on race (Latinos)

Wave 3	Hurricane Ian
(Intercept)	-1.11^{***} (0.33)
Female	-0.19 (0.11)
Evangelical	0.13 (0.16)
Democrat	-0.02 (0.13)
Republican	0.21 (0.15)
Highly Educated (6 categories)	-0.01 (0.04)
Age 18-34	-0.48^{*} (0.21)
Age 35-44	-0.16 (0.22)
Age 45-64	-0.37 (0.25)
Income (greater than $60k)	0.15 (0.12)
Born in the U.S.	0.46^{**} (0.14)
Cuban	-0.11 (0.24)
Mexican	-0.33^{*} (0.13)
Puerto Rican	0.21 (0.19)
Social Media Accounts	0.03 (0.02)
Social Media News Language (Bilingual)	-0.21 (0.18)
Social Media News Language (Spanish)	0.37 (0.22)
Bilingual home	0.10 (0.15)
Spanish home	0.22 (0.16)
Social Media Use (3 days or more)	-0.12 (0.16)
Fox News Viewer	0.09 (0.12)
Social Media News Language (Bilingual) * Social Media Use (3 days or more)	0.09 (0.24)
Social Media News Language (Spanish) * Social Media Use (3 days or more)	0.08 (0.29)
McFadden R^2	0.10
Percent Correctly Predicted	84.26%
AIC	773.80
BIC	884.79
Log Likelihood	-363.90
Deviance	727.80
Number of Observations	921

Note: the numbers between parenthesis represent standard errors. $^{***}p < 0.001$; $^{**}p < 0.01$; $^{*}p < 0.05$

Table C3: Probit Estimates for Wave 3 False Story on Venezuela purposely freeing inmates to send to the U.S. Border (Latinos)

Wave 3	Freeing Inmates
(Intercept)	-0.60 (0.29)*
Female	0.03 (0.10)
Evangelical	0.53 (0.15)***
Democrat	-0.21 (0.11)
Republican	0.25 (0.14)
Highly Educated (6 categories)	-0.04 (0.04)
Age 18-34	-0.64 (0.20)**
Age 35-44	-0.39 (0.21)
Age 45-64	-0.03 (0.22)
Income (greater than $60k)	0.25 (0.11)*
Born in the U.S.	0.25 (0.12)*
Cuban	0.05 (0.21)
Mexican	-0.34 (0.12)**
Puerto Rican	-0.07 (0.18)
Social Media Accounts	0.00 (0.02)
Social Media News Language (Bilingual)	-0.13 (0.16)
Social Media News Language (Spanish)	0.03 (0.21)
Bilingual home	0.34 (0.13)*
Spanish home	0.40 (0.14)**
Social Media Use (3 days or more)	0.05 (0.15)
Fox News Viewer	0.07 (0.11)
Social Media News Language (Bilingual) * Social Media Use (3 days or more)	0.22 (0.21)
Social Media News Language (Spanish) * Social Media Use (3 days or more)	0.55 (0.28)
McFadden R^2	0.12
Percent Correctly Predicted	76.76%
AIC	965.44
BIC	1076.42
Log Likelihood	-459.72
Deviance	919.44
Number of Observations	921

Note: the numbers between parenthesis represent standard errors. $^{***}p < 0.001$; $^{**}p < 0.01$; $^{*}p < 0.05$

Table C4: Probit Estimates for Wave 3 False Story on the majority of Planned Parenthood Clinics closing down across the country. (Latinos)

Wave 3	Planned Parenthood
(Intercept)	−1.23 (0.29)***
Female	0.01 (0.10)
Evangelical	0.07 (0.15)
Democrat	0.22 (0.11)*
Republican	0.26 (0.14)
Highly Educated (6 categories)	−0.06 (0.04)
Age 18-34	−0.35 (0.20)
Age 35-44	−0.25 (0.21)
Age 45-64	−0.21 (0.23)
Income (greater than $60k)	0.08 (0.11)
Born in the U.S.	0.66 (0.13)***
Cuban	0.25 (0.21)
Mexican	−0.31 (0.12)**
Puerto Rican	−0.14 (0.18)
Number of Social Media Accounts	0.04 (0.02)*
Social Media News Language (Bilingual)	−0.01 (0.16)
Social Media News Language (Spanish)	0.41 (0.21)*
Bilingual home	0.16 (0.13)
Spanish home	0.23 (0.14)
Social Media Use (3 days or more)	0.30 (0.14)*
Fox News Viewer	−0.14 (0.11)
Social Media News Language (Bilingual) * Social Media Use (3 days or more)	−0.12 (0.21)
Social Media News Language (Spanish) * Social Media Use (3 days or more)	−0.28 (0.28)
McFadden R^2	0.07
Percent Correctly Predicted	76.33%
AIC	984.69
BIC	1095.68
Log Likelihood	−469.35
Deviance	938.69
Number of Observations	921

Note: the numbers between parenthesis represent standard errors. $^{***}p < 0.001$; $^{**}p < 0.01$; $^{*}p < 0.05$

Table C5: Probit Estimates for Wave 3 False Story on that getting the COVID-19 Vaccine can make breast milk dangerous to infants. (Latinos)

Wave 3	COVID-19 Breast Milk
(Intercept)	−1.89 (0.39)***
Female	−0.12 (0.12)
Evangelical	0.32 (0.16)*
Democrat	−0.20 (0.14)
Republican	−0.05 (0.16)
Highly Educated (6 categories)	−0.00 (0.05)
Age 18-34	−0.03 (0.27)
Age 35-44	0.31 (0.28)
Age 45-64	0.18 (0.30)
Income (greater than $60k)	−0.04 (0.13)
Born in the U.S.	0.73 (0.16)***
Cuban	−0.48 (0.29)
Mexican	−0.40 (0.14)**
Puerto Rican	−0.38 (0.21)
Social Media Accounts	0.02 (0.03)
Social Media News Language (Bilingual)	0.12 (0.19)
Social Media News Language (Spanish)	0.36 (0.23)
Bilingual home	0.19 (0.16)
Spanish home	0.43 (0.17)**
Social Media Use (3 days or more)	0.11 (0.18)
Fox News Viewer	0.29 (0.13)*
Social Media News Language (Bilingual) * Social Media Use (3 days or more)	−0.40 (0.26)
Social Media News Language (Spanish) * Social Media Use (3 days or more)	0.18 (0.31)
McFadden R^2	0.13
Percent Correctly Predicted	87.62%
AIC	665.28
BIC	776.27
Log Likelihood	−309.64
Deviance	619.28
Number of Observations	921

Note: the numbers between parenthesis represent standard errors. $^{***}p < 0.001$; $^{**}p < 0.01$; $^{*}p < 0.05$

Table C6: Probit Estimates for Wave 5 False Story on U.S. aid to Ukraine was laundered back to the Democratic Party through the failed cryptocurrency exchange firm FTX. (Latinos)

Wave 5	Ukraine Aid
(Intercept)	−0.87 (0.33)**
Female	−0.01 (0.11)
Evangelical	0.40 (0.15)**
Democrat	−0.29 (0.12)*
Republican	0.10 (0.15)
Highly Educated (6 categories)	−0.09 (0.04)*
Age 18-34	−0.20 (0.23)
Age 35-44	−0.12 (0.24)
Age 45-64	−0.45 (0.27)
Income (greater than $60k)	0.33 (0.12)**
Born in the U.S.	0.27 (0.14)
Cuban	−0.15 (0.24)
Mexican	−0.20 (0.13)
Puerto Rican	0.09 (0.19)
Social Media Accounts	0.03 (0.02)
Social Media News Language (Bilingual)	−0.17 (0.17)
Social Media News Language (Spanish)	0.34 (0.21)
Bilingual home	−0.09 (0.15)
Spanish home	0.01 (0.15)
Social Media Use (3 days or more)	−0.06 (0.16)
Fox News Viewer	0.21 (0.12)
Social Media News Language (Bilingual) * Social Media Use (3 days or more)	0.13 (0.23)
Social Media News Language (Spanish) * Social Media Use (3 days or more)	0.36 (0.28)
McFadden R^2	0.09
Percent Correctly Predicted	83.54%
AIC	817.75
BIC	929.40
Log Likelihood	−385.88
Deviance	771.75
Number of Observations	948

Note: the numbers between parenthesis represent standard errors. $^{***}p < 0.001$; $^{**}p < 0.01$; $^{*}p < 0.05$

Table C7: Probit Estimates for Wave 5 False Story on voting sites in Arizona that experienced issues with tabulating ballots on Election Day during the 2022 Midterm Elections were conservative areas in Arizona's Maricopa County. (Latinos)

Wave 5	Maricopa Voting Sites
(Intercept)	−0.72 (0.29)*
Female	0.04 (0.09)
Evangelical	0.13 (0.15)
Democrat	−0.05 (0.10)
Republican	−0.11 (0.14)
Highly Educated (6 categories)	−0.09 (0.04)*
Age 18-34	−0.43 (0.19)*
Age 35-44	−0.53 (0.21)**
Age 45-64	−0.30 (0.22)
Income (greater than $60k)	0.10 (0.10)
Born in the U.S.	0.37 (0.12)**
Cuban	−0.05 (0.22)
Mexican	0.06 (0.11)
Puerto Rican	−0.01 (0.17)
Social Media Accounts	0.04 (0.02)
Social Media News Language (Bilingual)	−0.01 (0.15)
Social Media News Language (Spanish)	0.45 (0.20)*
Bilingual home	0.15 (0.13)
Spanish home	0.12 (0.13)
Social Media Use (3 days or more)	0.21 (0.11)
Fox News Viewer	0.19 (0.14)
Social Media News Language (Bilingual) * Social Media Use (3 days or more)	0.14 (0.20)
Social Media News Language (Spanish) * Social Media Use (3 days or more)	−0.04 (0.27)
McFadden R^2	0.04
Percent Correctly Predicted	75%
AIC	1075.70
BIC	1187.35
Log Likelihood	−514.85
Deviance	1029.70
Number of Observations	948

Note: the numbers between parenthesis represent standard errors. $^{***}p < 0.001$; $^{**}p < 0.01$; $^{*}p < 0.05$

Table C8: Probit Estimates for Wave 3 True Story on the U.S. government will provide $2 Million in aid to Cuba to support recovery efforts following Hurricane Ian (Latinos)

Wave 3	Foreign Aid to Cuba
(Intercept)	−0.07 (0.28)
Female	−0.29** (0.09)
Evangelical	0.11 (0.15)
Democrat	0.18 (0.10)
Republican	0.37** (0.14)
Highly Educated (6 categories)	0.04 (0.03)
Age 18-34	0.18 (0.19)
Age 35-44	−0.07 (0.20)
Age 45-64	−0.28 (0.22)
Income (greater than $60k)	0.22* (0.10)
Born in the U.S.	−0.05 (0.11)
Cuban	−0.04 (0.21)
Mexican	−0.13 (0.11)
Puerto Rican	−0.31 (0.18)
Social Media Accounts	−0.05* (0.02)
Social Media News Language (Bilingual)	−0.04 (0.14)
Social Media News Language (Spanish)	0.07 (0.20)*
Bilingual home	−0.10 (0.12)
Spanish home	−0.24 (0.13)
Social Media Use (3 days or more)	−0.35* (0.14)
Fox News Viewer	0.74*** (0.11)
Social Media News Language (Bilingual) * Social Media Use (3 days or more)	0.15 (0.19)
Social Media News Language (Spanish) * Social Media Use (3 days or more)	0.79** (0.28)
McFadden R^2	0.14
Percent Correctly Predicted	70.68%
AIC	1131.49
BIC	1242.48
Log Likelihood	−542.75
Deviance	1085.49
Number of Observations	921

Note: the numbers between parenthesis represent standard errors. $^{***}p < 0.001$; $^{**}p < 0.01$; $^{*}p < 0.05$

Table C9: Probit Estimates for Wave 3 True Story about the Department of Homeland Security expanding Title 42 expulsion in order to turn away Venezuelans that present at the United States-Mexico Border seeking asylum (Latinos)

Wave 3	Venezuelans Asylum Ban
(Intercept)	−0.13 (0.28)
Female	−0.33*** (0.09)
Evangelical	0.01 (0.15)
Democrat	0.19 (0.10)
Republican	0.35** (0.13)
Highly Educated (6 categories)	0.01 (0.03)
Age 18-34	0.10 (0.19)
Age 35-44	−0.04 (0.20)
Age 45-64	0.01 (0.22)
Income (greater than $60k)	0.27* (0.10)
Born in the U.S.	−0.19 (0.11)
Cuban	−0.31 (0.21)
Mexican	−0.14 (0.11)
Puerto Rican	−0.29 (0.18)
Social Media Accounts	−0.09*** (0.02)
Social Media News Language (Bilingual)	0.00 (0.14)
Social Media News Language (Spanish)	−0.05 (0.20)*
Bilingual home	0.32** (0.12)
Spanish home	0.43** (0.13)
Social Media Use (3 days or more)	0.326 (0.14)
Fox News Viewer	0.62*** (0.10)
Social Media News Language (Bilingual) * Social Media Use (3 days or more)	−0.17 (0.19)
Social Media News Language (Spanish) * Social Media Use (3 days or more)	0.07 (0.28)
McFadden R^2	0.13
Percent Correctly Predicted	68.29%
AIC	1151.94
BIC	1262.93
Log Likelihood	−552.97
Deviance	1105.94
Number of Observations	921

Note: the numbers between parenthesis represent standard errors. $^{***}p < 0.001$; $^{**}p < 0.01$; $^{*}p < 0.05$

Table C10: Probit Estimates for Wave 3 True Story on President Biden promising that if the Democrats retain control of the House and Senate, the first bill he will send to Congress will federally protect abortion rights (Latinos)

Wave 3	Abortion Rights
(Intercept)	−0.32 (0.28)
Female	−0.25** (0.09)
Evangelical	−0.04 (0.15)
Democrat	0.45*** (0.10)
Republican	0.39** (0.14)
Highly Educated (6 categories)	0.13*** (0.03)
Age 18-34	−0.05 (0.19)
Age 35-44	−0.20 (0.20)
Age 45-64	−0.30 (0.22)
Income (greater than $60k)	0.25** (0.10)
Born in the U.S.	0.013 (0.11)
Cuban	−0.09 (0.21)
Mexican	0.06 (0.11)
Puerto Rican	−0.11 (0.18)
Social Media Accounts	−0.03* (0.02)
Social Media News Language (Bilingual)	0.16 (0.14)
Social Media News Language (Spanish)	0.03 (0.20)*
Bilingual home	−0.15 (0.12)
Spanish home	−0.13 (0.13)
Social Media Use (3 days or more)	0.06 (0.14)
Fox News Viewer	0.17 (0.11)
Social Media News Language (Bilingual) * Social Media Use (3 days or more)	−0.05 (0.19)
Social Media News Language (Spanish) * Social Media Use (3 days or more)	0.09 (0.28)
McFadden R^2	0.08
Percent Correctly Predicted	66.78%
AIC	1143.74
BIC	1254.23
Log Likelihood	−548.62
Deviance	1097.24
Number of Observations	921

Note: the numbers between parenthesis represent standard errors. $^{***}p < 0.001$; $^{**}p < 0.01$; $^{*}p < 0.05$

Table C11: Probit Estimates for Wave 3 True Story on a rare correlation between the COVID-19 vaccine and Myocarditis, an inflammation of the heart muscle that can cause chest pain and shortness of breath, especially in young men (Latinos)

Wave 3	COVID-19 and Myocarditis
(Intercept)	-0.62^{*} (0.27)
Female	0.07 (0.09)
Evangelical	0.12 (0.15)
Democrat	0.05 (0.10)
Republican	0.22 (0.13)
Highly Educated (6 categories)	0.11^{**} (0.03)
Age 18-34	0.23 (0.19)
Age 35-44	0.25 (0.20)
Age 45-64	−0.00 (0.21)
Income (greater than $60k)	0.17 (0.10)
Born in the U.S.	−0.08 (0.11)
Cuban	0.06 (0.21)
Mexican	0.03 (0.11)
Puerto Rican	−0.07 (0.18)
Social Media Accounts	−0.01 (0.02)
Social Media News Language (Bilingual)	0.02 (0.14)
Social Media News Language (Spanish)	0.28 (0.20)*
Bilingual home	0.05 (0.12)
Spanish home	-0.26^{*} (0.13)
Social Media Use (3 days or more)	0.19 (0.14)
Fox News Viewer	0.35^{***} (0.11)
Social Media News Language (Bilingual) * Social Media Use (3 days or more)	−0.16 (0.19)
Social Media News Language (Spanish) * Social Media Use (3 days or more)	−0.13 (0.27)
McFadden R^2	0.06
Percent Correctly Predicted	62.54%
AIC	1231.52
BIC	1342.50
Log Likelihood	−592.76
Deviance	1085.52
Number of Observations	921

Note: the numbers between parenthesis represent standard errors. $^{***}p < 0.001$; $^{**}p < 0.01$; $^{*}p < 0.05$

Table C12: Probit Estimates for Wave 3 True Story about New York Representative George Santos made numerous dubious and false claims about his biography, work history, and financial status while running for office (Latinos)

Wave 5	George Santos claims
(Intercept)	0.14 (0.28)
Female	−0.28** (0.09)
Evangelical	−0.23 (0.15)
Democrat	0.43*** (0.10)
Republican	0.44** (0.14)
Highly Educated (6 categories)	0.05 (0.03)
Age 18-34	0.01 (0.19)
Age 35-44	−0.25 (0.20)
Age 45-64	−0.05 (0.22)
Income (greater than $60k)	0.20* (0.10)
Born in the U.S.	−0.12 (0.11)
Cuban	−0.31 (0.21)
Mexican	−0.09 (0.11)
Puerto Rican	0.21 (0.18)
Social Media Accounts	−0.02 (0.02)
Social Media News Language (Bilingual)	0.02 (0.14)
Social Media News Language (Spanish)	−0.46* (0.20)*
Bilingual home	0.03 (0.12)
Spanish home	−0.23 (0.13)
Social Media Use (3 days or more)	0.26 (0.14)
Fox News Viewer	0.12 (0.11)
Social Media News Language (Bilingual) * Social Media Use (3 days or more)	−0.11 (0.19)
Social Media News Language (Spanish) * Social Media Use (3 days or more)	0.08 (0.28)
McFadden R^2	0.08
Percent Correctly Predicted	68.25%
AIC	1186.12
BIC	1297.77
Log Likelihood	−570.06
Deviance	1140.12
Number of Observations	948

Note: the numbers between parenthesis represent standard errors. $^{***}p < 0.001$; $^{**}p < 0.01$; $^{*}p < 0.05$

APPENDIX D: Robustness Checks

We conduct a series of robustness checks to our Spanish-language social media first difference calculations for false and true political narratives. We first test to see if other politically relevant factors affect the probability of believing in false narratives. First, we calculate the difference in predicted probabilities between Latino respondents who identify as Republicans and those who identify as Democrats (Figures D1 and D2). We find differences in only 2 out of 7 false political narratives, notably, both related to foreign affairs: Venezuela freeing inmates, and Ukraine foreign aid through cryptocurrency. For true political narratives, there are no differences among Latino respondents in terms of partisan identity. Next, we compare the first differences between Latino respondents that report watching Fox News in comparison to those who do not (Figures D3 and D4). We find that Fox News viewers are more likely to believe in only 2 out of 7 false political narratives (i.e., Social Security and COVID-19 Breast Milk), and less likely to believe that Planned Parenthood clinics are shutting down across the country in comparison to those respondents who report not to watch Fox News.

Since we are fundamentally interested in a binary outcome "does a respondent believe the story" we estimated and reported probit models for the main section of the paper. But here we conduct a series of robustness checks for for model specification as well as variable measurement (i.e., coding of our outcome variable) for the results reported in the paper.

First, we estimated the model using OLS to see if our inferences were sensitive to model specification. We specified the model exactly as the probit models were specified, except the dependent variable now ranged from 1 ("very sure is true") to 5 ("very sure is false").[2] In Figure D5 and Figure D6 we present first differences analogous to Figures 1 and 2 in the paper.[3] We see the same result: respondents who used Spanish language social media were more likely to perceive six of the seven false statements as true.[4] Using Spanish language social media only made respondents more likely to perceive one of the true statements to be true.

Second, we considered whether including respondent fixed effects would change our result. Results in the body of the paper are based on estimates of a separate model for responses *to each*

[2] For one question responses ranged form 1 ("definitely true") to 7 ("definitely false").

[3] Full model results are presented in Tables D1 thru D12.

[4] The size of the effect appears different as it is now on a scale from 1 to 5 rather than a probability ranging from 0 to 1, but the interpretation is the same.

question. Here, we estimate a pooled respondent-level fixed-effects probit model with a specification otherwise identical to those in the body of the paper, but including individual-level fixed effects and pooled over responsess to all questions. These results are reported in Table D13. The pooled specification assumes that using Spanish language social media would have the same effect on the probability of believing each of the seven false narratives are true. This model confirms the results in the paper, with the estimated effect of using Spanish language social media being 0.20 (confidence interval of 0.11 to 0.31). Estimating the same pooled model without fixed effects (Table D14) produces similar estimates (0.15, confidence interval of 0.10 to 0.19)).

Next, we considered whether the decision to recode the responses – where respondents could choose on a scale ranging from definitely true to definitely false – into a binary variable in the way we did influenced the results. In addition to estimating the OLS models described above, we recoded the outcome variable so that only responses of "very sure is false" were considered to be belief in the narrative (i.e., we did *not* treat "somewhat sure is false" as believing the story to be false) and re-estimated the same probit models that the results in the paper are based on, and computed first-differences.[5] The first differences from these models are reported in Figure D7 and Figure D8. We again see that consuming Spanish-language social media for news resulted in greater belief in false narratives. Only one estimate suggesting Spanish-language social media may have contributed to increased belief in true narratives is significant at the 95% level.

As an additional robustness check of variable coding, we recoded the responses to treat "unsure" as missing data rather than as a 'failure to believe true'. Estimated first-differences for these models are reported in Figure D9 and Figure D10. We again see the same result: that consuming social media in Spanish makes respondents more likely to believe false narratives (the magnitude of the effects appears somewhat larger with this measurement). Conversely, there are no noticeable effects on the true stories which remain virtually the same.[6]

[5]For the one question labeled slightly differently, we only coded "definitely false" as false.

[6]Full model estimates for both models of variable measurement change are reported in Tables D15 thru D26

Figure D1: First Difference Estimates for False Political Narratives (Party ID)

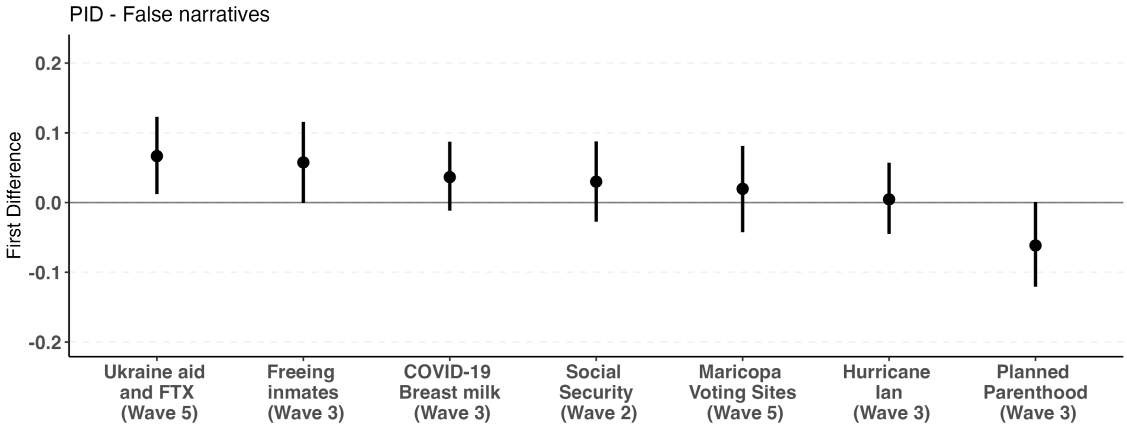

Note: The first difference in predicted probabilities between Latino respondents who identify as Republican and those who identify as Democrat are estimated from probit coefficients of Tables C1-C7.

Figure D2: First Difference Estimates for True Political Narratives (Party ID)

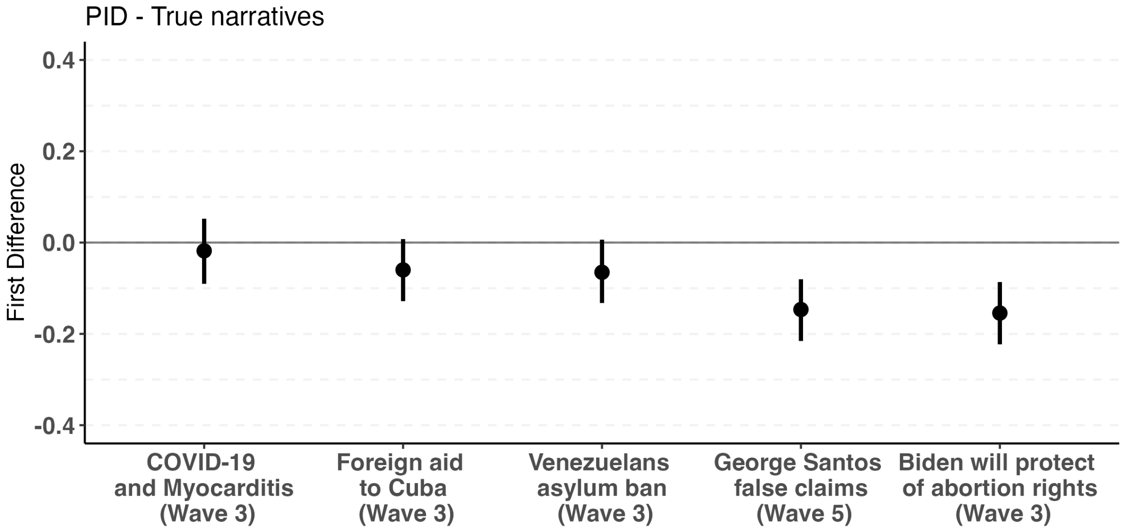

Note: The first difference in predicted probabilities between Latino respondents who identify as Republican and those who identify as Democrat are estimated from probit coefficients of Tables C8-C12.

Figure D3: First Difference Estimates for False Political Narratives (Watching Fox News)

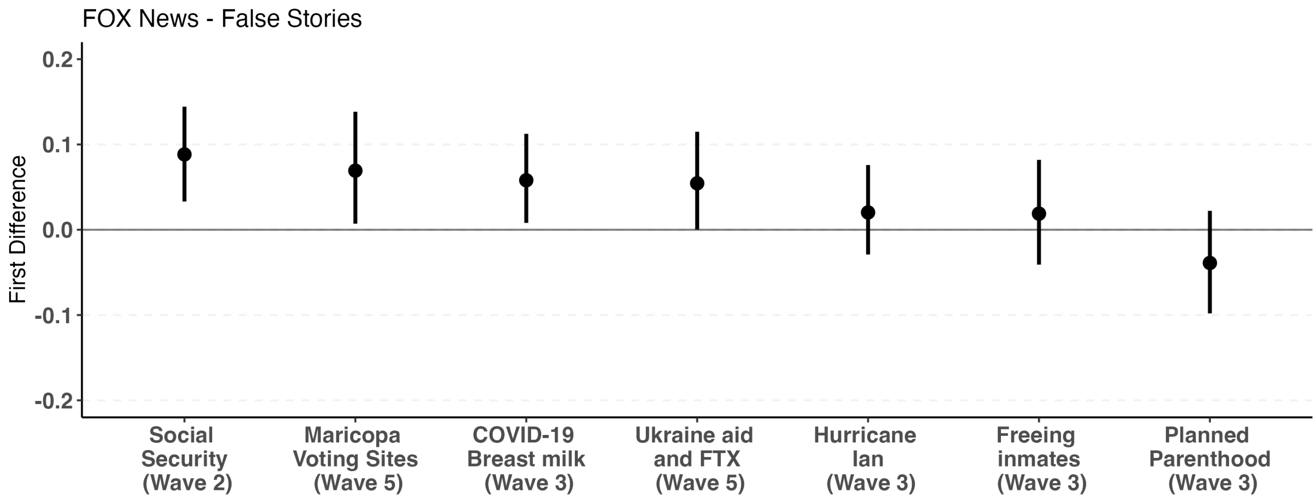

Note: The first difference in predicted probabilities between Latino respondents who watch Fox News and those who do not are estimated from probit coefficients of Tables C1-C7.

Figure D4: First Difference Estimates for True Political Narratives (Watching Fox News)

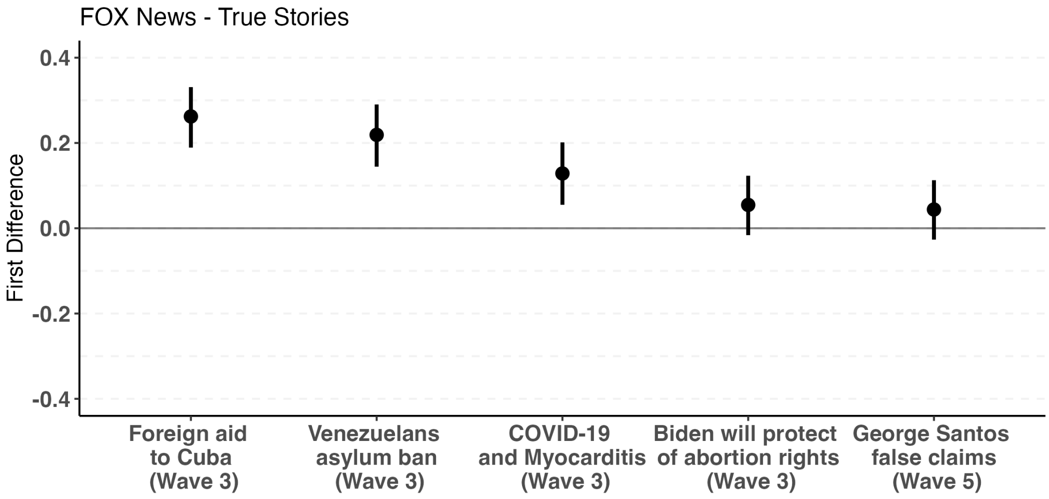

Note: The first difference in predicted probabilities between Latino respondents who watch Fox News and those who do not are estimated from probit coefficients of Tables C8-C12.

Figure D5: First Difference Estimates for False Political Narratives (OLS)

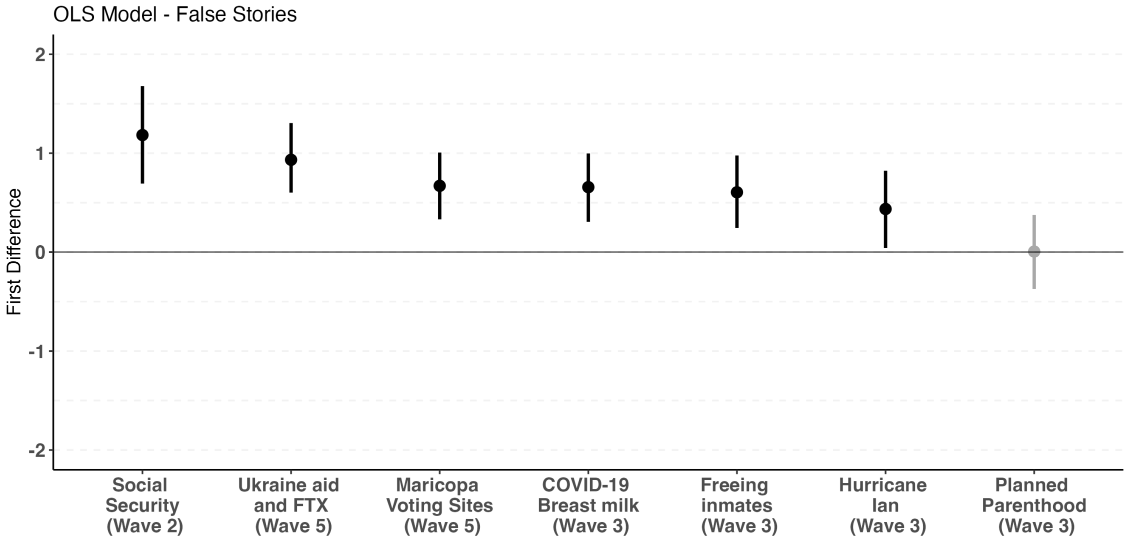

Note: First difference in predicted probabilities between Latino respondents who use Spanish-language social media and those who use English-language social media.

Figure D6: First Difference Estimates for True Political Narratives (OLS)

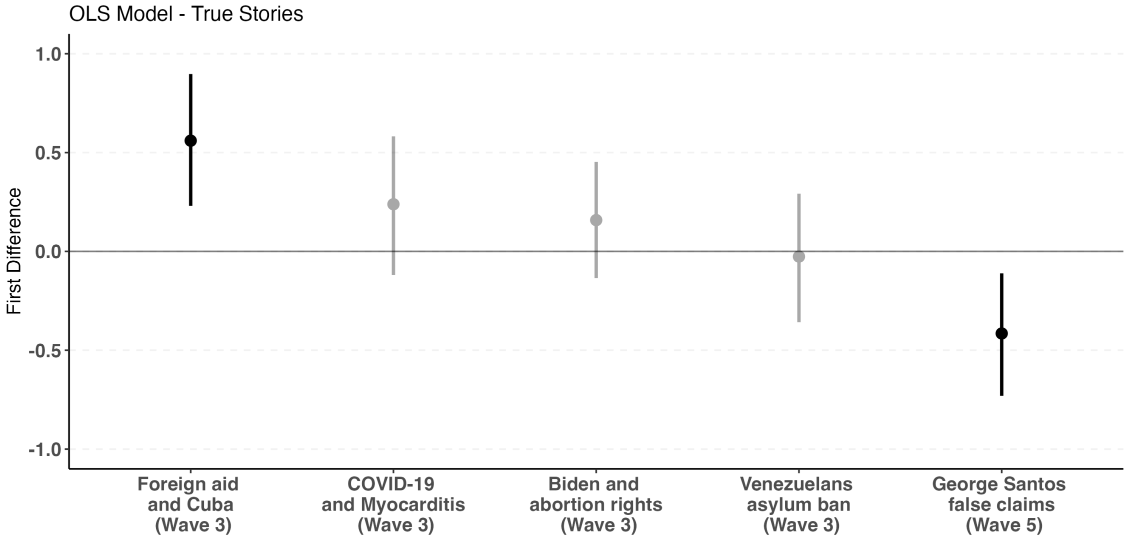

Note: First difference in predicted probabilities between Latino respondents who use Spanish-language social media and those who use English-language social media.

Figure D7: First Difference Estimates for False Political Narratives (confidently-held answers)

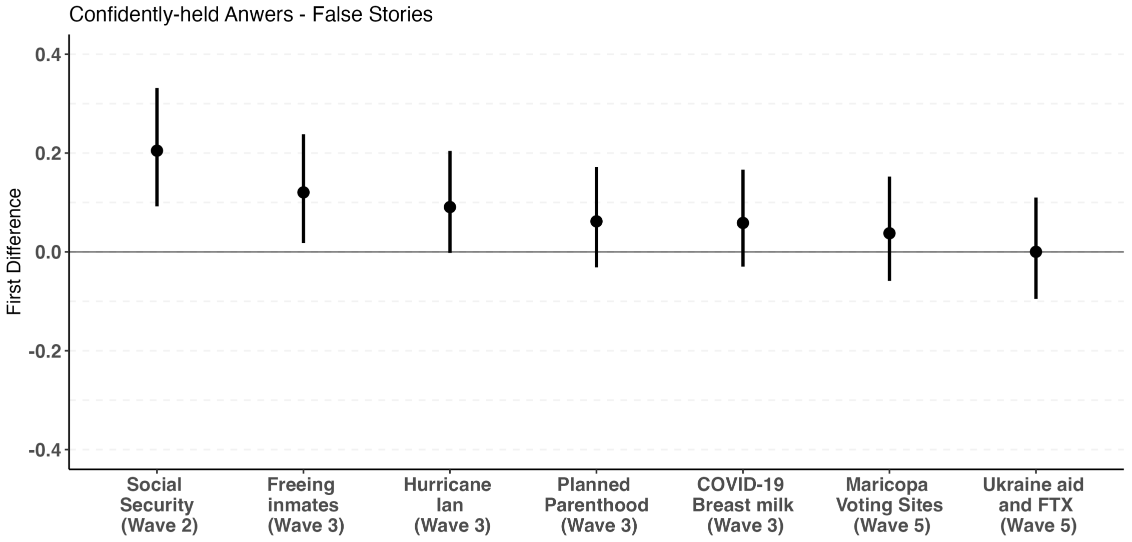

Note: The first difference in predicted probabilities between Latino respondents who use Spanish-language social media and those who use English-language social media are estimated from regression coefficients of Tables D1-D7. Confidently-held answers refer to exclusively "very sure is true" answers.

Figure D8: First Difference Estimates for True Political Narratives (confidently-held answers)

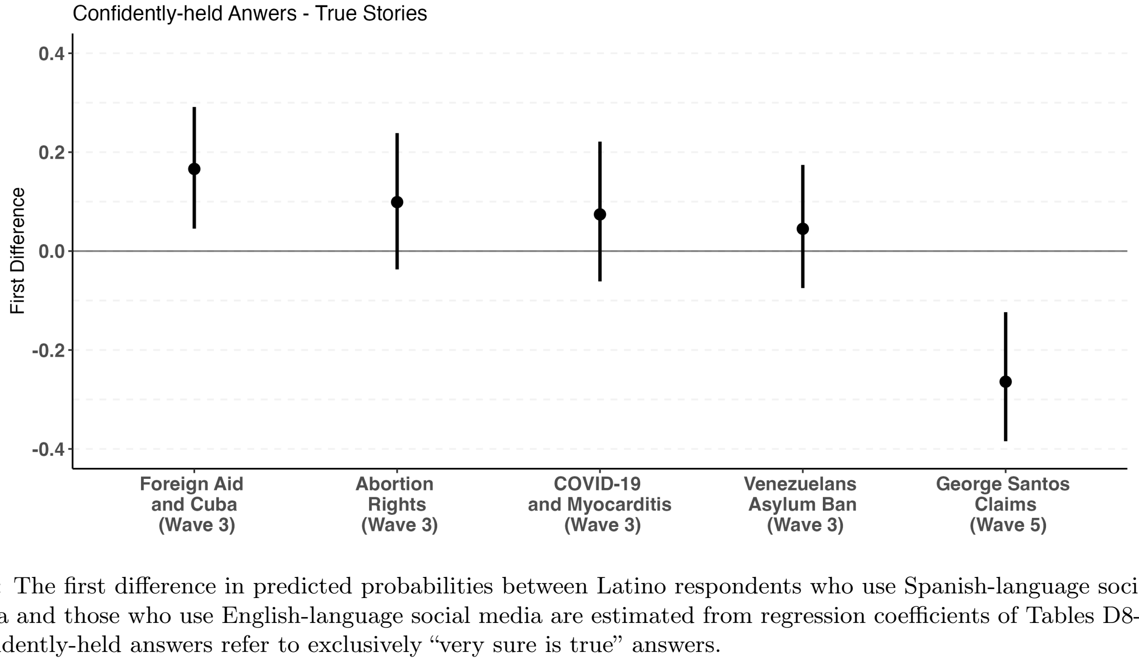

Note: The first difference in predicted probabilities between Latino respondents who use Spanish-language social media and those who use English-language social media are estimated from regression coefficients of Tables D8-D12. Confidently-held answers refer to exclusively "very sure is true" answers.

Figure D9: First Difference Estimates for False Political Narratives (without "Not sure" responses)

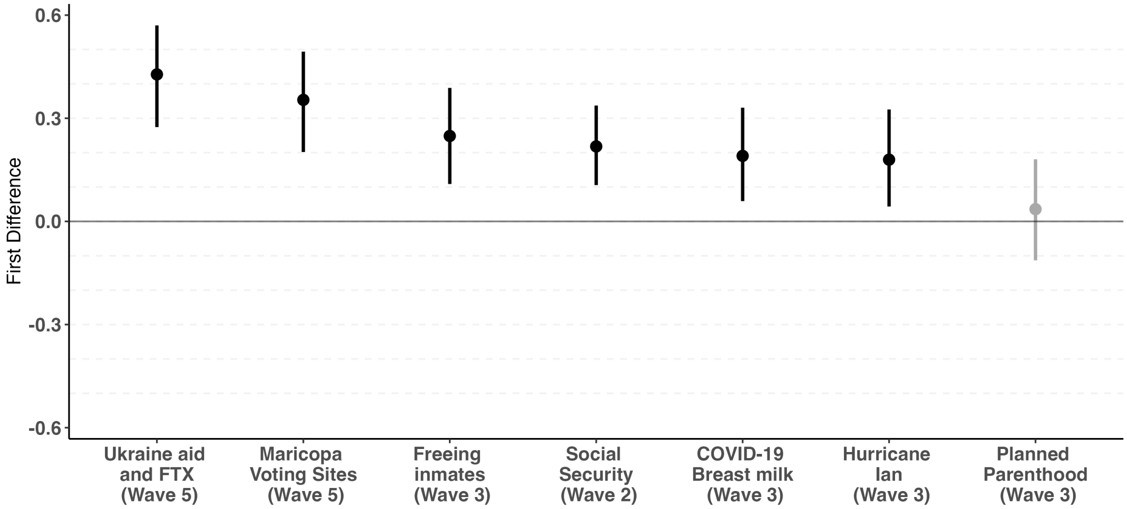

Note: The first difference in predicted probabilities between Latino respondents who use Spanish-language social media and those who use English-language social media are estimated from probit coefficients of Tables D1-D7. Respondents answering 'Don't Know' were treated as missing data and omitted from estimates.

Figure D10: First Difference Estimates for True Political Narratives (without "Not sure" responses)

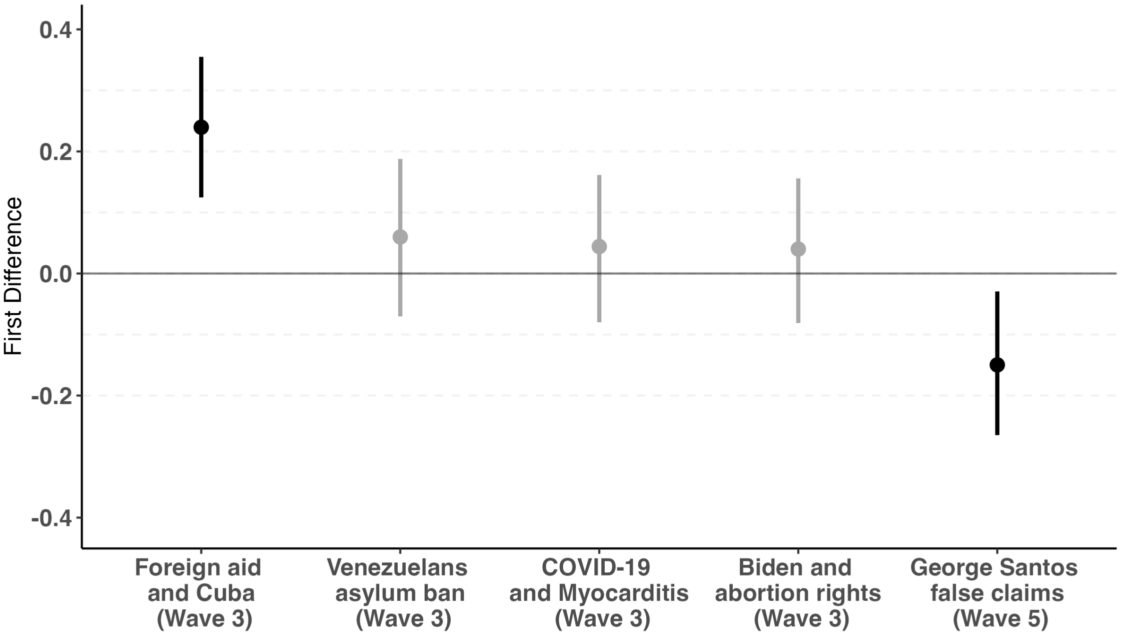

Note: The first difference in predicted probabilities between Latino respondents who use Spanish-language social media and those who use English-language social media are estimated from probit coefficients of Tables D8-D12. Respondents answering 'Don't Know' were treated as missing data and omitted from estimates.

Table D1: OLS Estimates for Wave 2 False Story on Social Security (Latinos)

	Social Security
Intercept	4.97 (0.33)***
Female	−0.13 (0.11)
Evangelical	−0.41 (0.18)*
Democrat	0.22 (0.12)
Republican	−0.27 (0.16)
Highly educated (6 categories)	0.16 (0.04)***
Age 18-34	0.08 (0.23)
Age 35-44	−0.46 (0.24)
Age 45-64	−0.32 (0.26)
Income (greater than $60k)	−0.01 (0.12)
Born in the U.S.	−0.97 (0.13)***
Cuban	0.55 (0.24)*
Mexican	0.67 (0.13)***
Puerto Rican	0.32 (0.21)
Number of Social Media Accounts	0.02 (0.02)
Social Media News Language (Bilingual)	0.03 (0.17)
Social Media News Language (Spanish)	−0.47 (0.24)*
Bilingual home	−0.12 (0.15)
Spanish home	−0.32 (0.16)*
Social Media Use (3 days or more)	0.21 (0.16)
Fox News Viewer	−0.28 (0.12)*
Social Media News Language (Bilingual) * Social Media Use (3 days or more)	−0.25 (0.23)
Social Media News Language (Spanish) * Social Media Use (3 days or more)	−0.71 (0.32)*
R^2	0.18
AIC	4338.70
BIC	4458.80
Number of Observations	1101

Note: the numbers between parenthesis represent standard errors. $^{***}p < 0.001$; $^{**}p < 0.01$; $^{*}p < 0.05$

Table D2: OLS Estimates for Wave 3 False Story on Hurricane Ian (Latinos)

	Hurricane Ian
Intercept	3.82 (0.25)***
Female	0.09 (0.08)
Evangelical	−0.27 (0.13)*
Democrat	0.17 (0.09)
Republican	−0.01 (0.12)
Highly educated (6 categories)	0.08 (0.03)**
Age 18-34	0.18 (0.18)
Age 35-44	−0.11 (0.19)
Age 45-64	0.20 (0.20)
Income (greater than $60k)	−0.03 (0.09)
Born in the U.S.	−0.50 (0.10)***
Cuban	−0.05 (0.19)
Mexican	0.24 (0.10)*
Puerto Rican	−0.40 (0.16)*
Number of Social Media Accounts	−0.01 (0.02)
Social Media News Language (Bilingual)	0.09 (0.13)
Social Media News Language (Spanish)	−0.31 (0.18)
Bilingual home	−0.24 (0.11)*
Spanish home	−0.38 (0.12)**
Social Media Use (3 days or more)	−0.02 (0.12)
Fox News Viewer	0.07 (0.10)
Social Media News Language (Bilingual) * Social Media Use (3 days or more)	0.08 (0.18)
Social Media News Language (Spanish) * Social Media Use (3 days or more)	−0.12 (0.25)
R^2	0.13
AIC	2985.03
BIC	3100.84
Number of Observations	921

Note: the numbers between parenthesis represent standard errors. $^{***}p < 0.001$; $^{**}p < 0.01$; $^{*}p < 0.05$

Table D3: OLS Estimates for Wave 3 False Story on Freeing Inmates (Latinos)

	Freeing Inmates
Intercept	3.15 (0.24)***
Female	−0.07 (0.08)
Evangelical	−0.50 (0.13)***
Democrat	0.15 (0.09)
Republican	−0.16 (0.12)
Highly educated (6 categories)	0.09 (0.03)**
Age 18-34	0.58 (0.17)***
Age 35-44	0.23 (0.18)
Age 45-64	−0.09 (0.19)
Income (greater than $60k)	0.02 (0.09)
Born in the U.S.	−0.28 (0.10)**
Cuban	−0.42 (0.18)*
Mexican	0.22 (0.09)*
Puerto Rican	−0.16 (0.15)
Number of Social Media Accounts	−0.03 (0.02)
Social Media News Language (Bilingual)	0.07 (0.13)
Social Media News Language (Spanish)	−0.20 (0.18)
Bilingual home	−0.22 (0.11)*
Spanish home	−0.34 (0.12)**
Social Media Use (3 days or more)	−0.08 (0.12)
Fox News Viewer	0.20 (0.09)*
Social Media News Language (Bilingual) * Social Media Use (3 days or more)	−0.14 (0.17)
Social Media News Language (Spanish) * Social Media Use (3 days or more)	−0.41 (0.24)
R^2	0.17
AIC	2912.62
BIC	3028.43
Number of Observations	921

Note: the numbers between parenthesis represent standard errors. $^{***}p < 0.001$; $^{**}p < 0.01$; $^{*}p < 0.05$

Table D4: OLS Estimates for Wave 3 False Story on Planned Parenthood (Latinos)

	Planned Parenthood
Intercept	3.54 (0.25)***
Female	−0.01 (0.08)
Evangelical	−0.25 (0.13)
Democrat	0.08 (0.09)
Republican	0.12 (0.12)
Highly educated (6 categories)	0.11 (0.03)***
Age 18-34	0.34 (0.17)*
Age 35-44	0.02 (0.18)
Age 45-64	0.00 (0.19)
Income (greater than $60k)	0.10 (0.09)
Born in the U.S.	−0.58 (0.10)***
Cuban	−0.33 (0.19)
Mexican	0.26 (0.09)**
Puerto Rican	0.01 (0.15)
Number of Social Media Accounts	−0.05 (0.02)**
Social Media News Language (Bilingual)	0.01 (0.13)
Social Media News Language (Spanish)	−0.49 (0.18)**
Bilingual home	−0.23 (0.11)*
Spanish home	−0.42 (0.12)***
Social Media Use (3 days or more)	−0.27 (0.12)*
Fox News Viewer	0.28 (0.09)**
Social Media News Language (Bilingual) * Social Media Use (3 days or more)	0.15 (0.17)
Social Media News Language (Spanish) * Social Media Use (3 days or more)	0.49 (0.24)*
R^2	0.15
AIC	2919.15
BIC	3034.97
Number of Observations	921

Note: the numbers between parenthesis represent standard errors. $^{***}p < 0.001$; $^{**}p < 0.01$; $^{*}p < 0.05$

Table D5: OLS Estimates for Wave 3 False Story on Covid-19 Breast Milk (Latinos)

	Covid-19 Breast Milk
Intercept	3.60 (0.23)***
Female	0.00 (0.08)
Evangelical	−0.41 (0.12)***
Democrat	0.33 (0.09)***
Republican	0.23 (0.11)*
Highly educated (6 categories)	0.08 (0.03)**
Age 18-34	0.34 (0.16)*
Age 35-44	0.03 (0.17)
Age 45-64	0.08 (0.18)
Income (greater than $60k)	0.17 (0.08)*
Born in the U.S.	−0.61 (0.09)***
Cuban	0.14 (0.17)
Mexican	0.18 (0.09)*
Puerto Rican	−0.05 (0.14)
Number of Social Media Accounts	0.00 (0.02)
Social Media News Language (Bilingual)	−0.01 (0.12)
Social Media News Language (Spanish)	−0.31 (0.17)
Bilingual home	−0.15 (0.10)
Spanish home	−0.20 (0.11)
Social Media Use (3 days or more)	−0.02 (0.11)
Fox News Viewer	−0.12 (0.09)
Social Media News Language (Bilingual) * Social Media Use (3 days or more)	0.17 (0.16)
Social Media News Language (Spanish) * Social Media Use (3 days or more)	−0.34 (0.23)
R^2	0.16
AIC	2807.54
BIC	2923.35
Number of Observations	921

Note: the numbers between parenthesis represent standard errors. $^{***}p < 0.001$; $^{**}p < 0.01$; $^{*}p < 0.05$

Table D6: OLS Estimates for Wave 5 False Story on Ukraine Aid (Latinos)

	Ukraine Aid
Intercept	3.49 (0.23)***
Female	−0.25 (0.07)***
Evangelical	−0.36 (0.12)**
Democrat	0.39 (0.08)***
Republican	0.06 (0.11)
Highly educated (6 categories)	0.12 (0.03)***
Age 18-34	0.35 (0.16)*
Age 35-44	0.17 (0.17)
Age 45-64	0.19 (0.18)
Income (greater than $60k)	−0.04 (0.08)
Born in the U.S.	−0.24 (0.09)**
Cuban	−0.00 (0.17)
Mexican	0.18 (0.08)*
Puerto Rican	−0.07 (0.14)
Number of Social Media Accounts	−0.05 (0.02)**
Social Media News Language (Bilingual)	0.01 (0.11)
Social Media News Language (Spanish)	−0.60 (0.16)***
Bilingual home	−0.08 (0.10)
Spanish home	−0.20 (0.10)
Social Media Use (3 days or more)	−0.00 (0.11)
Fox News Viewer	−0.06 (0.08)
Social Media News Language (Bilingual) * Social Media Use (3 days or more)	−0.07 (0.15)
Social Media News Language (Spanish) * Social Media Use (3 days or more)	−0.32 (0.22)
R^2	0.17
AIC	2844.19
BIC	2960.69
Number of Observations	948

Note: the numbers between parenthesis represent standard errors. $^{***}p < 0.001$; $^{**}p < 0.01$; $^{*}p < 0.05$

Table D7: OLS Estimates for Wave 5 False Story on Maricopa Voting Sites (Latinos)

	Maricopa Voting Sites
Intercept	$2.79\ (0.23)^{***}$
Female	$-0.16\ (0.07)^{*}$
Evangelical	$-0.24\ (0.12)^{*}$
Democrat	$0.18\ (0.08)^{*}$
Republican	$0.35\ (0.11)^{**}$
Highly educated (6 categories)	$0.12\ (0.03)^{***}$
Age 18-34	$0.51\ (0.16)^{**}$
Age 35-44	$0.50\ (0.16)^{**}$
Age 45-64	$0.35\ (0.18)$
Income (greater than $60k)	$-0.03\ (0.08)$
Born in the U.S.	$-0.07\ (0.09)$
Cuban	$-0.10\ (0.17)$
Mexican	$-0.02\ (0.08)$
Puerto Rican	$-0.12\ (0.14)$
Number of Social Media Accounts	$-0.05\ (0.02)^{**}$
Social Media News Language (Bilingual)	$-0.07\ (0.11)$
Social Media News Language (Spanish)	$-0.45\ (0.15)^{**}$
Bilingual home	$-0.16\ (0.10)$
Spanish home	$-0.08\ (0.10)$
Social Media Use (3 days or more)	$-0.03\ (0.11)$
Fox News Viewer	$-0.01\ (0.08)$
Social Media News Language (Bilingual) * Social Media Use (3 days or more)	$-0.09\ (0.15)$
Social Media News Language (Spanish) * Social Media Use (3 days or more)	$-0.20\ (0.21)$
R^2	0.11
AIC	2826.64
BIC	2943.15
Number of Observations	948

Note: the numbers between parenthesis represent standard errors. $^{***}p < 0.001$; $^{**}p < 0.01$; $^{*}p < 0.05$

Table D8: OLS Estimates for Wave 3 True Story on Foreign Aid to Cuba (Latinos)

	Foreign Aid to Cuba
Intercept	2.57 (0.22)***
Female	0.23 (0.07)**
Evangelical	−0.06 (0.12)
Democrat	−0.09 (0.08)
Republican	−0.25 (0.11)*
Education	−0.02 (0.03)
Age 18-34	−0.29 (0.15)
Age 35-44	−0.07 (0.16)
Age 45-64	0.07 (0.17)
Income (greater than $60k)	−0.13 (0.08)
Born in the U.S.	0.04 (0.09)
Cuban	0.14 (0.17)
Mexican	0.13 (0.08)
Puerto Rican	0.27 (0.14)*
Number of Social Media Accounts	0.06 (0.02)***
Social Media News Language (Bilingual)	−0.06 (0.11)
Social Media News Language (Spanish)	−0.10 (0.16)
Bilingual home	−0.04 (0.10)
Spanish home	0.16 (0.10)
Social Media Use (daily)	0.16 (0.11)
Fox News Viewer	−0.65 (0.08)***
Social Media News Language (Bilingual) * Social Media Use (daily)	0.03 (0.15)
Social Media News Language (Spanish) * Social Media Use (daily)	−0.46 (0.22)*
R^2	0.20
AIC	2718.36
BIC	2834.17
Number of Observations	921

Note: the numbers between parenthesis represent standard errors. $^{***}p < 0.001$; $^{**}p < 0.01$; $^{*}p < 0.05$

Table D9: OLS Estimates for Wave 3 True Story on Asylum Ban (Latinos)

	Asylum Ban
Intercept	2.68 (0.21)***
Female	0.26 (0.07)***
Evangelical	−0.02 (0.11)
Democrat	−0.09 (0.08)
Republican	−0.21 (0.10)*
Education	−0.04 (0.03)
Age 18-34	−0.35 (0.15)*
Age 35-44	−0.12 (0.16)
Age 45-64	−0.09 (0.17)
Income (greater than $60k)	−0.11 (0.08)
Born in the U.S.	0.19 (0.09)*
Cuban	0.10 (0.16)
Mexican	0.06 (0.08)
Puerto Rican	0.08 (0.13)
Number of Social Media Accounts	0.10 (0.02)***
Social Media News Language (Bilingual)	−0.03 (0.11)
Social Media News Language (Spanish)	0.08 (0.15)
Bilingual home	−0.42 (0.09)***
Spanish home	−0.37 (0.10)***
Social Media Use (daily)	−0.21 (0.10)*
Fox News Viewer	−0.45 (0.08)***
Social Media News Language (Bilingual) * Social Media Use (daily)	0.21 (0.15)
Social Media News Language (Spanish) * Social Media Use (daily)	−0.05 (0.21)
R^2	0.18
AIC	2664.21
BIC	2780.02
Number of Observations	921

Note: the numbers between parenthesis represent standard errors. $^{***}p < 0.001$; $^{**}p < 0.01$; $^{*}p < 0.05$

Table D10: OLS Estimates for Wave 3 True Story on Abortion Rights (Latinos)

	Abortion Rights
Intercept	2.40 (0.19)***
Female	0.20 (0.06)**
Evangelical	0.11 (0.10)
Democrat	−0.31 (0.07)***
Republican	−0.28 (0.09)**
Education	−0.09 (0.02)***
Age 18-34	0.05 (0.13)
Age 35-44	0.21 (0.14)
Age 45-64	0.23 (0.15)
Income (greater than $60k)	−0.17 (0.07)*
Born in the U.S.	0.01 (0.08)
Cuban	0.01 (0.15)
Mexican	−0.08 (0.07)
Puerto Rican	−0.00 (0.12)
Number of Social Media Accounts	0.04 (0.01)**
Social Media News Language (Bilingual)	−0.13 (0.10)
Social Media News Language (Spanish)	0.08 (0.14)
Bilingual home	0.10 (0.08)
Spanish home	0.09 (0.09)
Social Media Use (daily)	0.02 (0.09)
Fox News Viewer	−0.19 (0.07)**
Social Media News Language (Bilingual) * Social Media Use (daily)	−0.02 (0.13)
Social Media News Language (Spanish) * Social Media Use (daily)	−0.24 (0.19)
R^2	0.12
AIC	2478.00
BIC	2593.81
Number of Observations	921

Note: the numbers between parenthesis represent standard errors. $^{***}p < 0.001$; $^{**}p < 0.01$; $^{*}p < 0.05$

Table D11: OLS Estimates for Wave 5 True Story on Covid-19 Myocarditis (Latinos)

	Covid-19 Myocarditis
Intercept	2.66 (0.22)***
Female	0.05 (0.07)
Evangelical	0.00 (0.12)
Democrat	−0.01 (0.08)
Republican	−0.13 (0.11)
Education	−0.09 (0.03)***
Age 18-34	−0.13 (0.15)
Age 35-44	−0.13 (0.16)
Age 45-64	−0.00 (0.18)
Income (greater than $60k)	−0.06 (0.08)
Born in the U.S.	0.04 (0.09)
Cuban	−0.03 (0.17)
Mexican	0.04 (0.08)
Puerto Rican	0.00 (0.14)
Number of Social Media Accounts	0.06 (0.02)***
Social Media News Language (Bilingual)	−0.11 (0.11)
Social Media News Language (Spanish)	−0.22 (0.16)
Bilingual home	−0.09 (0.10)
Spanish home	0.23 (0.10)*
Social Media Use (daily)	−0.14 (0.11)
Fox News Viewer	−0.31 (0.08)***
Social Media News Language (Bilingual) * Social Media Use (daily)	0.22 (0.15)
Social Media News Language (Spanish) * Social Media Use (daily)	−0.01 (0.22)
R^2	0.10
AIC	2736.47
BIC	2852.28
Number of Observations	921

Note: the numbers between parenthesis represent standard errors. $^{***}p < 0.001$; $^{**}p < 0.01$; $^{*}p < 0.05$

Table D12: OLS Estimates for Wave 5 True Story on George Santos (Latinos)

	George Santos
Intercept	2.17 (0.21)***
Female	0.21 (0.07)**
Evangelical	0.23 (0.11)*
Democrat	−0.25 (0.07)***
Republican	−0.30 (0.10)**
Education	−0.03 (0.03)
Age 18-34	0.15 (0.14)
Age 35-44	0.29 (0.15)
Age 45-64	0.05 (0.17)
Income (greater than $60k)	−0.07 (0.07)
Born in the U.S.	−0.07 (0.08)
Cuban	0.24 (0.15)
Mexican	−0.05 (0.08)
Puerto Rican	−0.02 (0.12)
Number of Social Media Accounts	0.01 (0.01)
Social Media News Language (Bilingual)	−0.00 (0.10)
Social Media News Language (Spanish)	0.43 (0.14)**
Bilingual home	−0.08 (0.09)
Spanish home	0.08 (0.09)
Social Media Use (daily)	−0.34 (0.10)***
Fox News Viewer	0.11 (0.08)
Social Media News Language (Bilingual) * Social Media Use (daily)	0.17 (0.14)
Social Media News Language (Spanish) * Social Media Use (daily)	0.02 (0.20)
R^2	0.11
AIC	2661.45
BIC	2777.95
Number of Observations	948

Note: the numbers between parenthesis represent standard errors. $^{***}p < 0.001$; $^{**}p < 0.01$; $^{*}p < 0.05$

Table D13: Probit Model with Individual-level Fixed Effects (Latinos)

	Dependent variable:
	False Stories
Female	−0.006 (0.010)
Evangelical	0.077*** (0.016)
Democrat	−0.014 (0.011)
Republican	0.027* (0.015)
Highly Educated (6 categories)	−0.015*** (0.004)
Age 18-34	−0.075*** (0.021)
Age 35-44	−0.025 (0.022)
Age 45-64	−0.023 (0.024)
Income (greater than $60k)	0.041*** (0.011)
Born in the U.S.	0.130*** (0.012)
Cuban	−0.024 (0.023)
Mexican	−0.076*** (0.012)
Puerto Rican	−0.031 (0.019)
Number of Social Media Accounts	0.004 (0.002)
Social Media News Language (Bilingual)	−0.008 (0.016)
Social Media News Language (Spanish)	0.087*** (0.022)
Bilingual home	0.034** (0.013)
Spanish home	0.057*** (0.014)
Social Media Use (3 days or more)	0.012 (0.015)
Fox News Viewer	0.045*** (0.012)
Social Media News Language (Bilingual) * Social Media Use (3 days or more)	0.013 (0.021)
Social Media News Language (Spanish) * Social Media Use (3 days or more)	0.079*** (0.030)
Individual-level Fixed Effects	Yes ✓
McFadden R^2	0.07
AIC	6376.854
BIC	6533.415
Log. Likelihood	−3165.427
Number of Observations	6,681

Note: the numbers between parenthesis represent standard errors. $^{***}p < 0.001$; $^{**}p < 0.01$; $^{*}p < 0.05$

Table D14: Probit Model for Pooled False Narratives (Latinos)

	Dependent variable:
	False Stories
Intercept	−0.983*** (0.114)
Female	−0.022 (0.038)
Evangelical	0.238*** (0.056)
Democrat	−0.073* (0.043)
Republican	0.089* (0.054)
Highly Educated (6 categories)	−0.056*** (0.014)
Age 18-34	−0.279*** (0.077)
Age 35-44	−0.103 (0.081)
Age 45-64	−0.076 (0.088)
Income (greater than $60k)	0.141*** (0.041)
Born in the U.S.	0.481*** (0.048)
Cuban	−0.080 (0.082)
Mexican	−0.290*** (0.044)
Puerto Rican	−0.118* (0.068)
Number of Social Media Accounts	0.014* (0.008)
Social Media News Language (Bilingual)	−0.058 (0.061)
Social Media News Language (Spanish)	0.271*** (0.077)
Bilingual home	0.117** (0.050)
Spanish home	0.200*** (0.053)
Social Media Use (3 days or more)	0.044 (0.056)
Fox News Viewer	0.139*** (0.042)
Social Media News Language (Bilingual) * Social Media Use (3 days or more)	0.078 (0.080)
Social Media News Language (Spanish) * Social Media Use (3 days or more)	0.213** (0.104)
McFadden R^2	0.06
AIC	6,430.075
BIC	6,586.637
Log Likelihood	−3,192.038
Number of Observations	6,681

Note: the numbers between parenthesis represent standard errors. $^{***}p < 0.001$; $^{**}p < 0.01$; $^{*}p < 0.05$

Table D15: Probit Estimates for Wave 2 False Story on Social Security without "Not Sure" answers or only confidently-held answers (Latinos)

Wave 2 - Social Security	without "Not Sure"	only confidently-held
Intercept	-0.90 (0.32)**	-2.69 (0.49)***
Female	-0.00 (0.10)	0.03 (0.15)
Evangelical	0.42 (0.17)*	0.05 (0.22)
Democrat	-0.17 (0.11)	0.17 (0.18)
Republican	0.07 (0.14)	0.10 (0.22)
Highly educated (6 categories)	-0.10 (0.04)**	-0.01 (0.06)
Age 18-34	0.16 (0.23)	0.11 (0.33)
Age 35-44	0.56 (0.24)*	0.27 (0.33)
Age 45-64	0.52 (0.26)*	0.07 (0.37)
Income (greater than $60k)	0.06 (0.11)	-0.05 (0.16)
Born in the U.S.	0.83 (0.13)***	0.71 (0.21)***
Cuban	-0.39 (0.21)	-0.22 (0.34)
Mexican	-0.64 (0.12)***	-0.14 (0.18)
Puerto Rican	-0.34 (0.19)	-0.29 (0.27)
Number of Social Media Accounts	-0.03 (0.02)	-0.06 (0.03)
Social Media News Language (Bilingual)	0.00 (0.16)	-0.67 (0.41)
Social Media News Language (Spanish)	0.23 (0.21)	0.45 (0.31)
Bilingual home	0.10 (0.14)	0.50 (0.19)**
Spanish home	0.24 (0.14)	0.21 (0.22)
Social Media Use (3 days or more)	-0.14 (0.15)	0.50 (0.23)*
Fox News Viewer	0.32 (0.11)**	0.14 (0.16)
Social Media News Language (Bilingual) * Social Media Use (3 days or more)	0.22 (0.21)	0.79 (0.45)
Social Media News Language (Spanish) * Social Media Use (3 days or more)	0.48 (0.29)	0.53 (0.36)
McFadden R^2	0.16	0.20
Percent Correctly Predicted	75.9%	94.82%
AIC	971.81	415.95
BIC	1082.69	531.04
Log Likelihood	-462.90	-184.98
Deviance	925.81	369.95
Number of Observations	917	1,101

Note: The column "only confidently-held" codes the respondent as believing the story only if they answered "very sure is true."

The numbers between parenthesis represent standard errors. $^{***}p < 0.001$; $^{**}p < 0.01$; $^{*}p < 0.05$

Table D16: Probit Estimates for Wave 3 False Story on Hurricane Ian without "Not Sure" answers or only confidently-held answers (Latinos)

Wave 3 - Hurricane Ian	without "Not Sure"	only confidently-held
Intercept	-0.91^{**} (0.35)	-2.01^{***} (0.46)
Female	-0.14 (0.12)	-0.27 (0.15)
Evangelical	0.23 (0.18)	0.05 (0.21)
Democrat	-0.09 (0.14)	0.11 (0.18)
Republican	0.20 (0.17)	0.48^{*} (0.21)
Highly Educated (6 categories)	-0.06 (0.05)	0.00 (0.06)
Age 18-34	-0.37 (0.23)	-0.57 (0.29)
Age 35-44	-0.07 (0.24)	0.04 (0.29)
Age 45-64	-0.25 (0.27)	-0.23 (0.33)
Income (greater than $60k)	0.12 (0.13)	0.23 (0.16)
Born in the U.S.	0.53^{***} (0.16)	0.51^{*} (0.21)
Cuban	-0.04 (0.26)	-0.10 (0.34)
Mexican	-0.35^{*} (0.14)	0.00 (0.18)
Puerto Rican	0.40 (0.21)	0.21 (0.25)
Number of Social Media Accounts	0.02 (0.03)	-0.01 (0.03)
Social Media News Language (Bilingual)	-0.14 (0.20)	-0.75^{*} (0.32)
Social Media News Language (Spanish)	0.39 (0.23)	0.20 (0.29)
Bilingual home	0.22 (0.16)	0.43^{*} (0.20)
Spanish home	0.33 (0.17)	0.41 (0.22)
Social Media Use (3 days or more)	-0.04 (0.17)	-0.07 (0.22)
Fox News Viewer	0.03 (0.13)	0.25 (0.16)
Social Media News Language (Bilingual) * Social Media Use (3 days or more)	-0.08 (0.26)	0.68 (0.37)
Social Media News Language (Spanish) * Social Media Use (3 days or more)	0.21 (0.33)	0.35 (0.36)
McFadden R^2	0.12	0.19
Percent Correctly Predicted	81.89%	92.73%
AIC	685.41	416.98
BIC	790.67	527.96
Log Likelihood	-319.70	-185.49
Deviance	639.41	370.98
Number of Observations	718	921

Note: The column "only confidently-held" codes the respondent as believing the story only if they answered "very sure is true."

The numbers between parenthesis represent standard errors. $^{***}p < 0.001$; $^{**}p < 0.01$; $^{*}p < 0.05$

Table D17: Probit Estimates for Wave 3 False Story on Freeing Inmates without "Not Sure" answers or only confidently-held answers (Latinos)

Wave 3 - Freeing inmates	without "Not Sure"	only confidently-held
Intercept	−0.12 (0.35)	−1.70*** (0.42)
Female	0.08 (0.12)	0.01 (0.14)
Evangelical	0.68*** (0.18)	0.41* (0.19)
Democrat	−0.21 (0.13)	0.06 (0.17)
Republican	0.26 (0.16)	0.46* (0.19)
Highly Educated (6 categories)	−0.09 (0.05)	0.01 (0.05)
Age 18-34	−0.77** (0.25)	−0.16 (0.27)
Age 35-44	−0.43 (0.26)	−0.05 (0.28)
Age 45-64	0.15 (0.29)	−0.07 (0.30)
Income (greater than $60k)	0.10 (0.13)	−0.06 (0.15)
Born in the U.S.	0.34* (0.15)	−0.06 (0.17)
Cuban	0.29 (0.29)	0.20 (0.27)
Mexican	−0.37** (0.14)	−0.40* (0.18)
Puerto Rican	0.27 (0.24)	−0.02 (0.25)
Number of Social Media Accounts	0.01 (0.03)	−0.03 (0.03)
Social Media News Language (Bilingual)	−0.10 (0.19)	−0.20 (0.25)
Social Media News Language (Spanish)	0.40 (0.26)	0.01 (0.30)
Bilingual home	0.42** (0.16)	0.48* (0.19)
Spanish home	0.53** (0.17)	0.42* (0.20)
Social Media Use (3 days or more)	−0.05 (0.17)	0.13 (0.23)
Fox News Viewer	−0.01 (0.13)	−0.01 (0.15)
Social Media News Language (Bilingual) * Social Media Use (3 days or more)	0.26 (0.25)	0.38 (0.31)
Social Media News Language (Spanish) * Social Media Use (3 days or more)	0.37 (0.35)	0.62 (0.37)
McFadden R^2	0.17	0.12
Percent Correctly Predicted	72.81%	92.62%
AIC	738.07	476.92
BIC	840.69	587.91
Log Likelihood	−346.04	−215.46
Deviance	692.07	430.92
Number of Observations	640	921

Note: The column "only confidently-held" codes the respondent as believing the story only if they answered "very sure is true."

The numbers between parenthesis represent standard errors. $^{***}p < 0.001$; $^{**}p < 0.01$; $^{*}p < 0.05$

Table D18: Probit Estimates for Wave 3 False Story on Planned Parenthood without "Not Sure" answers or only confidently-held answers (Latinos)

Wave 3 - Planned Parenthood	without "Not Sure"	only confidently-held
Intercept	−0.62 (0.34)	−3.11*** (0.48)
Female	0.02 (0.12)	−0.09 (0.15)
Evangelical	0.10 (0.18)	0.28 (0.20)
Democrat	0.10 (0.13)	0.31 (0.18)
Republican	0.17 (0.16)	0.16 (0.22)
Highly Educated (6 categories)	−0.13** (0.04)	0.09 (0.06)
Age 18-34	−0.38 (0.23)	−0.33 (0.28)
Age 35-44	−0.14 (0.24)	−0.32 (0.30)
Age 45-64	−0.12 (0.27)	−0.51 (0.35)
Income (greater than $60k)	−0.03 (0.12)	−0.17 (0.16)
Born in the U.S.	0.66*** (0.14)	1.16*** (0.24)
Cuban	0.30 (0.25)	−0.08 (0.31)
Mexican	−0.37** (0.13)	−0.10 (0.18)
Puerto Rican	0.02 (0.21)	−0.23 (0.26)
Number of Social Media Accounts	0.05* (0.02)	−0.01 (0.03)
Social Media News Language (Bilingual)	0.05 (0.18)	0.06 (0.27)
Social Media News Language (Spanish)	0.64* (0.25)	0.71* (0.29)
Bilingual home	0.25 (0.15)	0.38* (0.19)
Spanish home	0.39* (0.16)	0.54** (0.20)
Social Media Use (3 days or more)	0.35* (0.16)	0.42 (0.23)
Fox News Viewer	−0.23 (0.13)	0.11 (0.16)
Social Media News Language (Bilingual) * Social Media Use (3 days or more)	−0.22 (0.24)	−0.14 (0.33)
Social Media News Language (Spanish) * Social Media Use (3 days or more)	−0.54 (0.34)	−0.35 (0.38)
McFadden R^2	0.10	0.15
Percent Correctly Predicted	70.62%	93.38%
AIC	783.80	426.09
BIC	886.41	537.07
Log Likelihood	−368.90	−190.04
Deviance	737.80	380.09
Number of Observations	640	921

Note: The column "only confidently-held" codes the respondent as believing the story only if they answered "very sure is true."

The numbers between parenthesis represent standard errors. $^{***}p < 0.001$; $^{**}p < 0.01$; $^{*}p < 0.05$

Table D19: Probit Estimates for Wave 3 False Story on COVID-19 Breast Milk without "Not Sure" answers or only confidently-held answers (Latinos)

Wave 3 - COVID-19 Breast Milk	without "Not Sure"	only confidently-held
Intercept	−1.52*** (0.44)	−3.22*** (0.61)
Female	−0.07 (0.13)	0.01 (0.18)
Evangelical	0.42* (0.19)	0.46* (0.23)
Democrat	−0.34* (0.15)	−0.10 (0.21)
Republican	−0.12 (0.18)	−0.27 (0.26)
Highly Educated (6 categories)	−0.03 (0.05)	0.14 (0.07)
Age 18-34	−0.06 (0.30)	−0.47 (0.33)
Age 35-44	0.32 (0.31)	−0.13 (0.34)
Age 45-64	0.28 (0.34)	−0.31 (0.38)
Income (greater than $60k)	−0.16 (0.14)	−0.23 (0.20)
Born in the U.S.	0.85*** (0.17)	0.61* (0.25)
Cuban	−0.53 (0.32)	−0.24 (0.40)
Mexican	−0.43** (0.16)	−0.22 (0.24)
Puerto Rican	−0.17 (0.25)	−0.01 (0.30)
Number of Social Media Accounts	−0.00 (0.03)	0.00 (0.04)
Social Media News Language (Bilingual)	0.18 (0.21)	−0.13 (0.39)
Social Media News Language (Spanish)	0.41 (0.25)	0.63 (0.35)
Bilingual home	0.24 (0.18)	0.41 (0.26)
Spanish home	0.45* (0.18)	0.76** (0.26)
Social Media Use (3 days or more)	0.15 (0.19)	0.62* (0.31)
Fox News Viewer	0.29* (0.14)	0.43* (0.19)
Social Media News Language (Bilingual) * Social Media Use (3 days or more)	−0.48 (0.28)	−0.42 (0.47)
Social Media News Language (Spanish) * Social Media Use (3 days or more)	0.27 (0.35)	−0.25 (0.44)
McFadden R^2	0.16	0.22
Percent Correctly Predicted	84.09%	96.09%
AIC	574.70	286.76
BIC	678.87	397.74
Log Likelihood	−264.35	−120.38
Deviance	528.70	240.76
Number of Observations	685	921

Note: The column "only confidently-held" codes the respondent as believing the story only if they answered "very sure is true."

The numbers between parenthesis represent standard errors. $^{***}p < 0.001$; $^{**}p < 0.01$; $^{*}p < 0.05$

Table D20: Probit Estimates for Wave 5 False Story on Ukraine Aid without "Not Sure" answers or only confidently-held answers (Latinos)

Wave 5 - Ukraine Aid	without "Not Sure"	only confidently-held
Intercept	−0.50 (0.38)	−3.15*** (0.67)
Female	0.12 (0.12)	−0.07 (0.21)
Evangelical	0.49** (0.18)	−0.00 (0.30)
Democrat	−0.44** (0.14)	0.02 (0.26)
Republican	0.01 (0.17)	0.72* (0.29)
Highly Educated (6 categories)	−0.12** (0.05)	−0.04 (0.09)
Age 18-34	−0.39 (0.26)	−0.40 (0.38)
Age 35-44	−0.27 (0.27)	−0.18 (0.39)
Age 45-64	−0.37 (0.31)	−0.31 (0.44)
Income (greater than $60k)	0.29* (0.13)	−0.23 (0.23)
Born in the U.S.	0.36* (0.16)	0.50 (0.30)
Cuban	−0.17 (0.28)	0.02 (0.40)
Mexican	−0.24 (0.14)	−0.34 (0.28)
Puerto Rican	0.09 (0.22)	0.38 (0.30)
Number of Social Media Accounts	0.03 (0.03)	0.05 (0.04)
Social Media News Language (Bilingual)	−0.11 (0.19)	−0.64 (0.50)
Social Media News Language (Spanish)	0.72** (0.25)	−4.31 (249.02)
Bilingual home	0.02 (0.16)	0.97*** (0.28)
Spanish home	0.25 (0.16)	0.76* (0.31)
Social Media Use (3 days or more)	−0.09 (0.17)	0.78* (0.32)
Fox News Viewer	0.18 (0.14)	0.31 (0.22)
Social Media News Language (Bilingual) * Social Media Use (3 days or more)	0.12 (0.26)	0.09 (0.56)
Social Media News Language (Spanish) * Social Media Use (3 days or more)	0.60 (0.36)	4.34 (249.02)
McFadden R^2	0.14	0.26
Percent Correctly Predicted	79.43%	97.05%
AIC	668.71	231.64
BIC	772.42	343.29
Log Likelihood	−311.36	−92.82
Deviance	622.71	185.64
Number of Observations	671	948

Note: The column "only confidently-held" codes the respondent as believing the story only if they answered "very sure is true."

The numbers between parenthesis represent standard errors. $^{***}p < 0.001$; $^{**}p < 0.01$; $^{*}p < 0.05$

Table D21: Probit Estimates for Wave 5 False Story on Maricopa Voting Sites without "Not Sure" answers or only confidently-held answers (Latinos)

Wave 5 - Maricopa Voting Sites	without "Not Sure"	only confidently-held
Intercept	0.34 (0.36)	−0.68 (0.46)
Female	0.14 (0.11)	−0.02 (0.17)
Evangelical	0.29 (0.19)	0.15 (0.24)
Democrat	−0.31* (0.13)	0.07 (0.20)
Republican	−0.39* (0.17)	0.38 (0.24)
Highly Educated (6 categories)	−0.15*** (0.04)	−0.24*** (0.07)
Age 18-34	−0.79** (0.25)	−0.70* (0.28)
Age 35-44	−0.84** (0.27)	−0.72* (0.30)
Age 45-64	−0.48 (0.29)	−0.69* (0.34)
Income (greater than $60k)	−0.02 (0.12)	0.03 (0.18)
Born in the U.S.	0.38** (0.14)	0.14 (0.21)
Cuban	−0.12 (0.26)	0.05 (0.35)
Mexican	0.07 (0.13)	0.04 (0.19)
Puerto Rican	0.16 (0.22)	−0.03 (0.30)
Number of Social Media Accounts	0.05* (0.02)	−0.03 (0.04)
Social Media News Language (Bilingual)	0.04 (0.18)	−0.13 (0.30)
Social Media News Language (Spanish)	0.68** (0.24)	0.32 (0.37)
Bilingual home	0.20 (0.15)	0.44* (0.22)
Spanish home	0.22 (0.16)	−0.01 (0.26)
Social Media Use (3 days or more)	0.08 (0.16)	0.44 (0.26)
Fox News Viewer	0.11 (0.13)	0.00 (0.19)
Social Media News Language (Bilingual) * Social Media Use (3 days or more)	0.17 (0.23)	0.00 (0.37)
Social Media News Language (Spanish) * Social Media Use (3 days or more)	0.35 (0.36)	−0.05 (0.45)
McFadden R^2	0.10	0.13
Percent Correctly Predicted	67.68%	95.89%
AIC	797.63	329.23
BIC	899.80	440.88
Log Likelihood	−375.81	−141.62
Deviance	751.63	283.23
Number of Observations	628	948

Note: The column "only confidently-held" codes the respondent as believing the story only if they answered "very sure is true."

The numbers between parenthesis represent standard errors. $^{***}p < 0.001$; $^{**}p < 0.01$; $^{*}p < 0.05$

Table D22: Probit Estimates without "Not Sure" answers or only confidently-held answers for Wave 3 True Story on Foreign aid to Cuba (Latinos)

Wave 3 - Foreign aid to Cuba	without "Not Sure"	only confidently-held
Intercept	0.00 (0.30)	−1.06** (0.35)
Female	−0.31** (0.10)	−0.37*** (0.11)
Evangelical	0.09 (0.16)	−0.13 (0.17)
Democrat	0.26* (0.11)	0.18 (0.13)
Republican	0.37* (0.14)	0.51*** (0.15)
Highly Educated (6 categories)	0.04 (0.04)	0.15*** (0.04)
Age 18-34	0.27 (0.20)	0.36 (0.23)
Age 35-44	−0.02 (0.22)	0.02 (0.24)
Age 45-64	−0.33 (0.23)	−0.42 (0.29)
Income (greater than $60k)	0.28** (0.10)	0.21 (0.11)
Born in the U.S.	−0.07 (0.12)	−0.23 (0.13)
Cuban	0.09 (0.24)	0.26 (0.23)
Mexican	−0.18 (0.11)	−0.21 (0.12)
Puerto Rican	−0.32 (0.19)	−0.72** (0.26)
Number of Social Media Accounts	−0.04* (0.02)	−0.12*** (0.02)
Social Media News Language (Bilingual)	−0.04 (0.15)	0.10 (0.17)
Social Media News Language (Spanish)	0.12 (0.22)	0.06 (0.23)
Bilingual home	−0.22 (0.13)	0.19 (0.14)
Spanish home	−0.31* (0.14)	−0.18 (0.16)
Social Media Use (3 days or more)	−0.32* (0.15)	−0.12 (0.16)
Fox News Viewer	0.70*** (0.11)	0.70*** (0.11)
Social Media News Language (Bilingual) * Social Media Use (3 days or more)	0.16 (0.21)	0.03 (0.23)
Social Media News Language (Spanish) * Social Media Use (3 days or more)	0.63* (0.30)	0.59 (0.31)
McFadden R^2	0.16	0.24
Percent Correctly Predicted	73.59%	81.76%
AIC	1000.05	820.77
BIC	1108.36	931.76
Log Likelihood	−477.02	−387.39
Deviance	954.05	774.77
Number of Observations	820	921

Note: The column "only confidently-held" codes the respondent as believing the story only if they answered "very sure is true."

The numbers between parenthesis represent standard errors. $^{***}p < 0.001$; $^{**}p < 0.01$; $^{*}p < 0.05$

Table D23: Probit Estimates without "Not Sure" answers or only confidently-held answers for Wave 3 True Story on Venezuelans Asylum Ban (Latinos)

Wave 3 - Venezuelans Asylum Ban	without "Not Sure"	only confidently-held
Intercept	0.07 (0.30)	-1.09^{**} (0.34)
Female	-0.35^{***} (0.10)	-0.36^{***} (0.11)
Evangelical	−0.04 (0.16)	−0.03 (0.17)
Democrat	0.27^{*} (0.11)	0.34^{**} (0.13)
Republican	0.40^{**} (0.15)	0.63^{***} (0.15)
Highly Educated (6 categories)	0.01 (0.04)	0.14^{***} (0.04)
Age 18-34	−0.01 (0.21)	0.39 (0.23)
Age 35-44	−0.12 (0.22)	0.15 (0.24)
Age 45-64	−0.05 (0.24)	0.12 (0.26)
Income (greater than $60k)	0.34^{**} (0.11)	0.12 (0.11)
Born in the U.S.	−0.17 (0.12)	-0.47^{***} (0.13)
Cuban	−0.25 (0.23)	−0.22 (0.25)
Mexican	-0.23^{*} (0.11)	-0.28^{*} (0.12)
Puerto Rican	-0.37^{*} (0.19)	−0.30 (0.22)
Number of Social Media Accounts	-0.07^{**} (0.02)	-0.15^{***} (0.02)
Social Media News Language (Bilingual)	0.04 (0.15)	0.05 (0.17)
Social Media News Language (Spanish)	0.01 (0.22)	−0.23 (0.23)
Bilingual home	0.17 (0.13)	0.44^{**} (0.14)
Spanish home	0.26 (0.14)	0.34^{*} (0.16)
Social Media Use (3 days or more)	0.18 (0.15)	0.09 (0.16)
Fox News Viewer	0.57^{***} (0.11)	0.49^{***} (0.11)
Social Media News Language (Bilingual) * Social Media Use (3 days or more)	−0.08 (0.21)	−0.08 (0.23)
Social Media News Language (Spanish) * Social Media Use (3 days or more)	0.17 (0.30)	0.40 (0.31)
McFadden R^2	0.17	0.20
Percent Correctly Predicted	74.70%	82.08%
AIC	1012.84	845.73
BIC	1120.79	956.71
Log Likelihood	−483.42	−399.86
Deviance	966.84	799.73
Number of Observations	807	921

Note: The column "only confidently-held" codes the respondent as believing the story only if they answered "very sure is true."

The numbers between parenthesis represent standard errors. $^{***}p < 0.001$; $^{**}p < 0.01$; $^{*}p < 0.05$

Table D24: Probit Estimates without "Not Sure" answers or only confidently-held answers for Wave 3 True Story on Abortion Rights (Latinos)

Wave 3 - Abortion Rights	without "Not Sure"	only confidently-held
Intercept	−0.24 (0.29)	−0.51 (0.29)
Female	−0.31** (0.10)	−0.41*** (0.10)
Evangelical	−0.06 (0.16)	−0.24 (0.16)
Democrat	0.39*** (0.11)	0.31** (0.11)
Republican	0.33* (0.14)	0.39** (0.14)
Highly Educated (6 categories)	0.13*** (0.04)	0.12** (0.04)
Age 18-34	−0.01 (0.20)	−0.16 (0.19)
Age 35-44	−0.11 (0.21)	−0.39 (0.20)
Age 45-64	−0.29 (0.23)	−0.36 (0.23)
Income (greater than $60k)	0.26** (0.10)	0.22* (0.10)
Born in the U.S.	0.16 (0.12)	−0.10 (0.12)
Cuban	−0.13 (0.21)	0.11 (0.22)
Mexican	0.07 (0.11)	0.10 (0.11)
Puerto Rican	−0.09 (0.18)	0.05 (0.18)
Number of Social Media Accounts	−0.02 (0.02)	−0.08*** (0.02)
Social Media News Language (Bilingual)	0.06 (0.15)	0.13 (0.15)
Social Media News Language (Spanish)	0.14 (0.22)	−0.00 (0.21)
Bilingual home	−0.14 (0.13)	−0.03 (0.13)
Spanish home	−0.15 (0.14)	−0.14 (0.14)
Social Media Use (3 days or more)	−0.02 (0.14)	−0.10 (0.14)
Fox News Viewer	0.25* (0.11)	0.34** (0.10)
Social Media News Language (Bilingual) * Social Media Use (3 days or more)	0.05 (0.20)	0.13 (0.20)
Social Media News Language (Spanish) * Social Media Use (3 days or more)	−0.00 (0.29)	0.32 (0.29)
McFadden R^2	0.08	0.10
Percent Correctly Predicted	90.81%	73.07%
AIC	1049.06	1079.11
BIC	1158.89	1190.10
Log Likelihood	−501.53	−516.56
Deviance	1003.06	1033.11
Number of Observations	876	921

Note: The column "only confidently-held" codes the respondent as believing the story only if they answered "very sure is true."

The numbers between parenthesis represent standard errors. $^{***}p < 0.001$; $^{**}p < 0.01$; $^{*}p < 0.05$

Table D25: Probit Estimates without "Not Sure" answers or only confidently-held answers for Wave 3 True Story on COVID-19 and Myocarditis (Latinos)

Wave 5 - COVID-19 and Myocarditis	without "Not Sure"	only confidently-held
Intercept	−0.73* (0.29)	−1.15*** (0.31)
Female	−0.07 (0.10)	−0.01 (0.10)
Evangelical	0.02 (0.15)	−0.11 (0.16)
Democrat	0.11 (0.11)	0.11 (0.11)
Republican	0.27 (0.14)	0.44** (0.14)
Highly Educated (6 categories)	0.12*** (0.04)	0.16*** (0.04)
Age 18-34	0.31 (0.20)	0.23 (0.21)
Age 35-44	0.27 (0.21)	0.12 (0.22)
Age 45-64	0.05 (0.23)	0.13 (0.24)
Income (greater than $60k)	0.20 (0.10)	−0.02 (0.11)
Born in the U.S.	−0.08 (0.12)	−0.19 (0.12)
Cuban	0.10 (0.22)	−0.23 (0.23)
Mexican	0.05 (0.11)	−0.20 (0.11)
Puerto Rican	−0.11 (0.18)	−0.22 (0.19)
Number of Social Media Accounts	0.01 (0.02)	−0.08*** (0.02)
Social Media News Language (Bilingual)	0.11 (0.15)	0.12 (0.15)
Social Media News Language (Spanish)	0.33 (0.21)	0.35 (0.21)
Bilingual home	−0.02 (0.13)	0.24 (0.13)
Spanish home	−0.28* (0.14)	−0.25 (0.15)
Social Media Use (3 days or more)	0.19 (0.14)	0.12 (0.15)
Fox News Viewer	0.29** (0.11)	0.39*** (0.11)
Social Media News Language (Bilingual) * Social Media Use (3 days or more)	−0.19 (0.20)	−0.39 (0.21)
Social Media News Language (Spanish) * Social Media Use (3 days or more)	−0.19 (0.29)	−0.12 (0.28)
McFadden R^2	0.07	0.12
Percent Correctly Predicted	80.03%	77.20%
AIC	1064.93	1009.62
BIC	1173.35	1120.60
Log Likelihood	−509.46	−481.81
Deviance	1018.93	963.62
Number of Observations	824	921

Note: The column "only confidently-held" codes the respondent as believing the story only if they answered "very sure is true."

The numbers between parenthesis represent standard errors. $^{***}p < 0.001$; $^{**}p < 0.01$; $^{*}p < 0.05$

Table D26: Probit Estimates without "Not Sure" answers or only confidently-held answers for Wave 5 True Story on George Santos claims (Latinos)

Wave 5 - George Santos Claims	without "Not Sure"	only confidently-held
Intercept	0.05 (0.30)	−0.32 (0.29)
Female	−0.37*** (0.10)	−0.41*** (0.09)
Evangelical	−0.24 (0.16)	−0.39* (0.16)
Democrat	0.46*** (0.11)	0.29** (0.11)
Republican	0.50*** (0.15)	0.49*** (0.14)
Highly Educated (6 categories)	0.08* (0.04)	0.06 (0.04)
Age 18-34	0.13 (0.21)	−0.50* (0.20)
Age 35-44	−0.13 (0.22)	−0.54** (0.21)
Age 45-64	0.01 (0.24)	−0.06 (0.23)
Income (greater than $60k)	0.23* (0.10)	−0.10 (0.10)
Born in the U.S.	−0.16 (0.12)	0.31** (0.12)
Cuban	−0.21 (0.23)	−0.18 (0.22)
Mexican	0.06 (0.11)	−0.01 (0.11)
Puerto Rican	0.29 (0.19)	−0.26 (0.18)
Number of Social Media Accounts	−0.01 (0.02)	−0.01 (0.02)
Social Media News Language (Bilingual)	0.05 (0.15)	0.02 (0.15)
Social Media News Language (Spanish)	−0.38 (0.21)	−0.55* (0.23)
Bilingual home	−0.11 (0.13)	−0.06 (0.12)
Spanish home	−0.33* (0.13)	−0.14 (0.13)
Social Media Use (3 days or more)	0.32* (0.15)	0.59*** (0.13)
Fox News Viewer	0.19 (0.12)	−0.42*** (0.11)
Social Media News Language (Bilingual) * Social Media Use (3 days or more)	−0.16 (0.21)	−0.21 (0.19)
Social Media News Language (Spanish) * Social Media Use (3 days or more)	−0.08 (0.28)	−0.34 (0.31)
McFadden R^2	0.11	0.14
Percent Correctly Predicted	87.50%	72.15%
AIC	1021.07	1091.45
BIC	1130.90	1203.10
Log Likelihood	−487.53	−522.73
Deviance	975.07	1045.45
Number of Observations	876	948

Note: The column "only confidently-held" codes the respondent as believing the story only if they answered "very sure is true."

The numbers between parenthesis represent standard errors. $^{***}p < 0.001$; $^{**}p < 0.01$; $^{*}p < 0.05$

Table D27: Weights Regression Coefficients

Model	*Coef.*	Std. Error	p-value
**False stories**			
Social Security	0.02	0.08	0.75
Hurricane Ian	−0.08	0.10	0.38
Freeing Inmates	0.22	0.21	0.31
Planned Parenthood	−0.12	0.21	0.56
Covid-19 Breast Milk	−0.40	0.26	0.12
Ukraine Aid and FTX	0.13	0.23	0.56
Maricopa Voting Sites	0.14	0.20	0.50
**True stories**			
Foreign Aid to Cuba	0.15	0.08	0.05
Venezuelans Asylum Ban	−0.08	0.08	0.29
Abortion Rights	0.25	0.08	0.00
COVID-19 and Myocarditis	−0.09	0.08	0.27
George Santos Claims	0.14	0.08	0.08

Note: This analysis includes a check to ensure that the applied weightings do not serve as a statistically significant predictor of the observed outcomes. This was achieved by incorporating the weightings directly into the models as a variable and testing for statistical significance.

APPENDIX E: Methodology for Selecting False Narratives

All False narratives were drawn from fact checks published by one of three sources: AP News, Univision, and Factchequeado (see below for the source of each story). All three sources are members of the Poynter Institute's International Fact Checking Network, which assures that members uphold standards of non-partisanship, transparency, and methodology. We chose to draw our stories from professional fact checkers, because they provided an indication that the stories were popular enough to merit coverage by a publication. For example, Univision notes that two of their most important factors in selecting narratives to fact check is whether the piece of information has gone viral and if a person (or persons) that shared it have significant influence and reach.

Wave 2 False Narrative Question

Question Label: Social Security

Dates of Response Collection: 22 September 2022 - 22 October 2022

Question Text: Some people are claiming that U.S. border patrol agents have been giving out social security numbers to immigrants who cross into the United States at the Southern Border without authorization. How truthful do you think this claim is?

1. Definitely true
2. Very Likely true
3. Probably true
4. Not sure
5. Probably false
6. Very Likely false
7. Definitely false

Source: `https://apnews.com/article/fact-check-social-security-number-border-552180846074`

Wave 3 False Narrative Questions

Question Label: Hurricane Ian

Dates of Response Collection: 28 October 2022 - 08 November 2022

Question Text: Vice President Kamala Harris said that Hurricane Ian relief will be distributed based on race, with communities of color receiving aid first.

1. Very sure is true
2. Somewhat sure is true
3. Not sure
4. Somewhat sure is false
5. Very sure is false

Source: `https://www.univision.com/noticias/falso-kamala-harris-ayuda-huracan-ian-color-piel-rick-`

Question Label: Freeing Inmates

Dates of Response Collection: 28 October 2022 - 08 November 2022

Question Text: The Department of Homeland Security has confirmed that Venezuela is purposely freeing inmates and sending them to the United States-Mexico Border.

1. Very sure is true
2. Somewhat sure is true
3. Not sure
4. Somewhat sure is false
5. Very sure is false

Source: `https://www.univision.com/noticias/no-hay-evidencias-venezuela-prisiones-exconvictos-trum`

Question Label: Planned Parenthood

Dates of Response Collection: 28 October 2022 - 08 November 2022

Question Text: After the Dobbs vs. Jackson Women's Health decision that overturned the right to have an abortion, the majority of Planned Parenthood Clinics have closed down across the country.

1. Very sure is true
2. Somewhat sure is true
3. Not sure
4. Somewhat sure is false
5. Very sure is false

Source: `https://www.univision.com/noticias/falso-planned-parenthood-cerro-centros-por-anulacion-r`

Question Label: COVID-19 Breast Milk

Dates of Response Collection: 28 October 2022 - 08 November 2022

Question Text: A new study shows that getting the COVID-19 Vaccine can make breast milk dangerous to infants.

1. Very sure is true
2. Somewhat sure is true
3. Not sure
4. Somewhat sure is false
5. Very sure is false

Source: `https://www.univision.com/noticias/nuevo-estudio-no-comprueba-leche-materna-peligrosa`

Wave 5 False Narrative Questions

Question Label: Maricopa Voting Sites

Dates of Response Collection: 08 February 2023 - 23 October 2023

Question Text: The only voting sites in Arizona that experienced issues with tabulating ballots on Election Day during the 2022 Midterm Elections were conservative areas in Arizona's Maricopa County.

1. Definitely true
2. Probably true
3. Not sure
4. Probably false
5. Definitely false

Source: https://www.univision.com/noticias/falso-maquinas-votacion-fallaron-distritos-republicano

Question Label: Ukraine and FTX

Dates of Response Collection: 08 February 2023 - 23 October 2023

Question Text: U.S. aid to Ukraine was laundered back to the Democratic Party through the failed cryptocurrency exchange firm FTX.

1. Definitely true
2. Probably true
3. Not sure
4. Probably false
5. Definitely false

Source: https://factchequeado.com/verificaciones/20230103/ftx-ucrania-dinero-estados-unidos/

Wave 3 True Narrative Questions

Question Label: Title 42

Dates of Response Collection: 28 October 2022 - 08 November 2022

Question Text: The Department of Homeland Security will expand Title 42 expulsion in order to turn away Venezuelans that present at the United States-Mexico Border seeking asylum.

1. Very sure is true
2. Somewhat sure is true
3. Not sure
4. Somewhat sure is false
5. Very sure is false

Source: `https://www.nbcnews.com/politics/immigration/biden-administration-admit-venezuelan-migran`

Question Label: Abortion Rights

Dates of Response Collection: 28 October 2022 - 08 November 2022

Question Text: President Biden has promised that if the Democrats retain control of the House and Senate, the first bill he will send to Congress next year will federally protect abortion rights.

1. Very sure is true
2. Somewhat sure is true
3. Not sure
4. Somewhat sure is false
5. Very sure is false

Source: `https://apnews.com/article/abortion-2022-midterm-elections-biden-health-congress-f3ffadd`

Question Label: COVID-19 and Myocarditis

Dates of Response Collection: 28 October 2022 - 08 November 2022

Question Text: Some studies published in peer-review journals have observed a rare correlation between the COVID-19 vaccine and Myocarditis, an inflammation of the heart muscle that can cause chest pain and shortness of breath, especially in young men.

1. Very sure is true
2. Somewhat sure is true
3. Not sure
4. Somewhat sure is false
5. Very sure is false

Source: `https://www.nature.com/articles/s41569-021-00662-w`

Question Label: Foreign Aid to Cuba

Dates of Response Collection: 28 October 2022 - 08 November 2022

Question Text: The U.S. Government will provide $2 Million in aid to Cuba to support recovery efforts following Hurricane Ian.

1. Very sure is true
2. Somewhat sure is true
3. Not sure
4. Somewhat sure is false
5. Very sure is false

Source: `https://www.nbcnews.com/news/latino/rare-move-us-offers-aid-cuba-help-hurricane-ian-reco`[illegible]

Wave 5 True Narrative Question

Question Label: George Santos Claims

Dates of Response Collection: 08 February 2023 - 23 October 2023

Question Text: Recently elected New York Representative George Santos made numerous dubious and false claims about his biography, work history, and financial status while running for office.

1. Definitely true
2. Probably true
3. Not sure
4. Probably false
5. Definitely false

Source: `https://www.nbcnewyork.com/news/politics/list-of-george-santos-lies-all-the-mistruths-the`
`4043980/`

Social Media Questions (Wave 1)

Question Label: Social Media News Language

Dates of Response Collection: 04 March 2022 - 25 July 2022

Question Text: When you get your news from social media is it in English, Spanish or both?

1. English
2. Spanish
3. Both
4. Neither

Question Label: Social Media Use (3 days or more)

Dates of Response Collection: 04 March 2022 - 25 July 2022

Question Text: Now, thinking about your online habits: How often do you do each of these actions on social media websites?

"Click on links to read political news stories"

1. Everyday
2. 3-5 days a week
3. 1-2 days a week
4. Every few weeks
5. Less often
6. Never
7. Don't Know

Question Label: Social Media Accounts

Dates of Response Collection: 04 March 2022 - 25 July 2022

Question Text: Do you use any of the following social media sites? (check all that apply):

1. Twitter
2. Facebook
3. Instagram
4. Reddit
5. LinkedIn
6. GroupMe
7. Snapchat
8. YouTube
9. TikTok
10. WhatsApp
11. Telegram
12. Gab
13. Gettr
14. Parler
15. Other

Demographic Questions (Wave 1)

Question Label: Democrat/Republican

Dates of Response Collection: 04 March 2022 - 25 July 2022

Question Text: Generally speaking, do you usually think of yourself as a Republican, a Democrat, an Independent, or something else?

1. Republican
2. Democrat
3. Independent
4. Other
5. No preference

Question Label: Bilingual Home/Spanish Home

Dates of Response Collection: 04 March 2022 - 25 July 2022

Question Text: What language do you speak the most at home? Only Spanish, more Spanish than English, both equally, more English than Spanish, or only English?

1. Only Spanish
2. More Spanish than English
3. Both equally
4. More English than Spanish
5. Only English

Question Label: Born in the U.S.

Dates of Response Collection: 04 March 2022 - 25 July 2022

Question Text: Were you born in the United States (not including Puerto Rico), the island of Puerto Rico, or in another country?

1. United States
2. Puerto Rico
3. Another country

Question Label: Hispanic/White

Dates of Response Collection: 04 March 2022 - 25 July 2022

Question Text: What racial or ethnic group best describes you?

1. White
2. Black
3. Hispanic or Latino/a/x
4. Asian
5. Native American or Alaska Native
6. Middle Eastern or Northern African
7. Two or more races
8. Other
9. Prefer not to say

Question Label: Cuban/Mexican/Puerto Rican

Dates of Response Collection: 04 March 2022 - 25 July 2022

Question Text: Families of [HISPANIC DENOMINATION] origin or background in the United States come from many different countries. From which country does most of your family come from?

1. Argentina
2. Barbados
3. Belize
4. Bolivia
5. Brazil
6. Chile
7. Colombia
8. Costa Rica
9. Cuba
10. Dominican Republic
11. Ecuador
12. El Salvador
13. Falkland Islands
14. Guatemala
15. Guyana
16. Haiti
17. Honduras
18. Mexico
19. Nicaragua
20. Panama
21. Paraguay
22. Peru
23. Portugal
24. Puerto Rico
25. Spain
26. Suriname
27. Uruguay
28. Venezuela
29. French Guiana
30. Jamaica
31. Trinidad

Question Label: Income (greater than $60k)

Dates of Response Collection: 04 March 2022 - 25 July 2022

Question Text: Information about income is very important to understand. Would you please give your best guess? Please indicate the answer that includes your entire household income in (previous year) before taxes.

1. Less than $10,000
2. $10,000 to $19,999
3. $20,000 to $29,999
4. $30,000 to $39,999
5. $40,000 to $49,999
6. $50,000 to $59,999
7. $60,000 to $69,999
8. $70,000 to $79,999
9. $80,000 to $89,999
10. $90,000 to $99,999
11. $100,000 to $149,999
12. $149,999 or more
13. Prefer not to say

Question Label: Highly Educated

Dates of Response Collection: 04 March 2022 - 25 July 2022

Question Text: What is the highest level of education you have completed?

1. No high school degree
2. High school graduate
3. Some college, but no degree (yet)
4. 2-year college degree
5. 4-year college degree
6. Postgraduate degree

Question Label: Female

Dates of Response Collection: 04 March 2022 - 25 July 2022

Question Text: What is your gender?

1. Male
2. Female
3. Non-binary

Question Label: Age 18-34/Age 35-44/Age 45-64

Dates of Response Collection: 04 March 2022 - 25 July 2022

Question Text: How old are you?

1. Under 18
2. 18-24
3. 25-34 years old
4. 35-44 years old
5. 45-54 years old
6. 55-64 years old
7. 65+ years old

Question Label: Fox News Viewer

Dates of Response Collection: 04 March 2022 - 25 July 2022

Question Text: Do you use any of the following as sources for political news and information? Check all that apply:

1. Parler
2. Newsmax
3. Fox News
4. Breitbart
5. Infowars
6. None of these

Question Label: Evangelical

Dates of Response Collection: 04 March 2022 - 25 July 2022

Question Text: What is your religious preference?

1. Protestant
2. Evangelical or born-again christian
3. Roman Catholic
4. Mormon
5. Orthodox Church
6. Islam/Muslim
7. Jewish
8. Other Religion (Candomblé, Umbanda, Voodoo, Rastafarian, Mayan Traditional)
9. Other Religion/Christian
10. Other Religion/Non-Christian
11. No religion, Atheist, Agnostic
12. Prefer not to say

References

AAPOR. 2023. “Standard Definitions: Final Dispositions of Case Codes and Outcome Rates for Surveys.” *The American Association for Public Opinion Research* https://aapor.org/wp-content/uploads/2023/05/Standards-Definitions-10th-edition.pdf.

Ahler, Douglas J., Carolyn E. Roush, and Gaurav Sood. 2019. “The Micro-Task Market for Lemons: Data Quality on Amazon’s Mechanical Turk.” *Political Science Research and Methods* pp. 1–20.

Antoun, Christopher, Chengli Zhang, Frederick G. Conrad, and Michael F. Schober. 2016. “Comparisons of Online Recruitment Strategies for Convenience Samples: Craigslist, Google AdWords, Facebook, and Amazon Mechanical Turk.” *Field Methods* 28(3):231–246.

Boas, Thomas C., David P. Christenson, and David M. Glick. 2020. “Recruiting Large Online Samples in the United States and India: Facebook, Mechanical Turk, and Qualtrics.” *Political Science Research and Methods* 8(2):232–250.

Brown, Anna. 2015. “The unique challenges of surveying U.S. Latinos.” *Pew Researcg Center* https://www.pewresearch.org/methods/2015/11/12/the-unique-challenges-of-surveying-u-s-latinos/.

Bunge, Eduardo L., Lesley A Taylor, Melissa Bond, Taylor N. Stephens, Kara Nishimuta, Alinne Z. Barrera, Robert Wickham, , and Ricardo F. Muñoz. 2017. “Facebook for recruiting Spanish- and English-speaking smokers.” *Internet Interventions* 17:405–408.

Griffin, M., R.J. Martino, and C. LoSchiavo. 2022. “Ensuring Survey Research Data Integrity in the Era of Internet Bots.” *Qual Quant* 56:2841–2852.

Meta. 2022. “Preparing for Upcoming Removal of Certain Ad Targeting Options.” `https://www.facebook.com/government-nonprofits/blog/preparing-for-upcoming-removal-of-certain-ad-targeting-options`.

Neundorf, Anja, and Aykut Öztürk. 2023. “How to Improve Representativeness and Cost-Cffectiveness in Samples Recruited Through Meta: A Comparison of Advertisement Tools.” *PLoS ONE* 18(2):e0281243.

Schneider, Daniel, and Kristen Harknett. 2022. "What's to Like? Facebook as a Tool for Survey Data Collection." *Sociological Methods & Research* 51(1):108–140.

Sha, Mandy, Georgina McAvinchey, Rosanna Quiroz, and Jennifer Moncada. 2017. "Successful Techniques to Recruit Hispanic and Latino Research Participants." *Survey Practice* 10(3).

Trejo, Y.G., M. Meyers, M. Martinez, A. O'Brien, P. Goerman, and B.O. Class. 2022. "Identifying Data Quality Challenges in Online Opt-In Panels Using Cognitive Interviews in English and Spanish." *Journal of Official Statistics* 38(3):793–822.

Waggoner, P.D., R. Kennedy, and S. Clifford. 2019. "Detecting Fraud in Online Surveys by Tracing, Scoring, and Visualizing IP Addresses." *Journal of Open Source Software* 4(37):1285.

Zhang, Baobao, Matto Mildenberger, Peter D. Howe, Jennifer Marlon, Seth A. Rosenthal, and Anthony Leiserowitz. 2020. "Quota Sampling Using Facebook Advertisements." *Political Science Research and Methods* 8(3):558–564.
